# Prognostic Value of the KELIM Score Versus Conventional CA-125 Kinetics in Ovarian Cancer: A Systematic Review and Meta-Analysis

**DOI:** 10.3390/diagnostics16213474

**Published:** 2026-10-24

**Authors:** Ying-Hsi Chen, Dah-Ching Ding

**Affiliations:** 1 Department of Obstetrics and Gynecology, Hualien Tzu Chi Hospital, Buddhist Tzu Chi Medical Foundation, Hualien 970, Taiwan; 2 Institute of Medical Sciences, Tzu Chi University, Hualien 970, Taiwan

**Keywords:** KELIM, CA-125, ovarian cancer, chemosensitivity, cytoreduction, meta-analysis

## Abstract

**Background/Objectives**: To compare the prognostic value of the modeled cancer antigen-125 (CA-125) elimination rate constant K (KELIM) with that of conventional CA-125 kinetics (e.g., half-life) in epithelial ovarian cancer. **Methods**: PubMed, EMBASE, and Cochrane CENTRAL were searched from inception to 8 July 2026, using terms for ovarian cancer, CA-125 kinetics (KELIM, half-life, nadir), platinum-based chemotherapy, and survival or cytoreduction. Hazard ratios (HRs) and odds ratios (ORs) were pooled using random-effects models. **Results**: Thirty-seven studies were included (KELIM, 29; conventional, 8). Favorable KELIM was associated with longer overall survival (HR: 0.54, 95% confidence interval [CI]: 0.47–0.63; 14 cohorts; I^2^ = 72%; moderate certainty) and progression-free survival (HR: 0.57, 95% CI: 0.51–0.63; 18 cohorts; I^2^ = 62%) and with complete cytoreduction (OR: 3.34, 95% CI: 2.00–5.55; nine cohorts). Short CA-125 half-life was associated with longer overall survival (HR: 0.48, 95% CI: 0.38–0.60; three cohorts; low certainty); no study assessed both markers in the same cohort. Sensitivity analyses changed the estimates little. **Conclusions**: KELIM is a consistent prognostic factor in ovarian cancer. Its similarity to CA-125 half-life rests on an indirect comparison with older cohorts and does not establish equivalence or superiority. Evidence that KELIM predicts treatment benefit remains exploratory.

## 1. Introduction

Epithelial ovarian cancer (EOC) remains the most lethal gynecological malignancy, with approximately 75% of patients diagnosed with advanced FIGO stage III–IV disease [1]. First-line management involves a combination of platinum-based chemotherapy and cytoreductive surgery—in particular, primary cytoreductive surgery (PCS) or, increasingly, neoadjuvant chemotherapy (NACT) with interval cytoreductive surgery. Treatment success depends jointly on the completeness of surgical resection and the intrinsic chemosensitivity of the tumor [2].

For decades, cancer antigen-125 (CA-125), encoded by *MUC16*, has been recognized as the principal serum biomarker for EOC [3]. Conventional CA-125 parameters, including the baseline value, nadir, half-life, time to nadir, and the Gynecologic Cancer InterGroup (GCIG) response criterion, have all been evaluated as prognostic indicators; however, the GCIG criterion in particular has shown limited and inconsistent value [4].

The modeled CA-125 elimination rate constant K (KELIM) quantifies the kinetics of CA-125 decline within the first 100 days of chemotherapy using a semi-mechanistic model and requires three or more CA-125 measurements during the first three cycles [4,5]. The individual KELIM value is a rate constant expressed per day; because its absolute value varies with the treatment setting and population, it is standardized by dividing it by a reference value, either the median KELIM of a reference population or a previously optimized cut-off [6,7]; in the GCIG meta-analysis, for example, this value was 0.06 per day [6]. A standardized KELIM of 1.0 therefore corresponds to a CA-125 elimination rate equal to this reference value. Values ≥1.0 (favorable score) indicate faster-than-reference CA-125 decline, reflecting higher tumor chemosensitivity, and have been associated with longer progression-free and overall survival and a higher likelihood of complete cytoreduction, whereas values <1.0 (unfavorable score) indicate slower decline and lower chemosensitivity [6,7]. KELIM is therefore a continuous prognostic marker dichotomized at a conventional threshold and validated against clinical outcomes, rather than a diagnostic test with an external reference standard; some studies have applied alternative cut-offs, such as tercile-based categories [4]. Large, randomized trials and a GCIG individual-patient-data (IPD) meta-analysis have validated KELIM as an independent prognostic biomarker in advanced ovarian cancer [6]. A previous meta-analysis of the literature published before February 2023 pooled KELIM and conventional CA-125 parameters across primary and recurrent disease [8]. However, most KELIM studies, including the GCIG individual-patient-data meta-analysis, large national registries, and poly(ADP-ribose) polymerase (PARP) inhibitor era cohorts, have been published since; complete cytoreduction has not been synthesized as an outcome; and the evidence on KELIM-guided treatment selection has not been appraised with respect to whether it is prognostic or predictive.

To address these gaps, the present systematic review aimed to evaluate the prognostic value of KELIM and to compare it with conventional CA-125 kinetic parameters; its association with surgical outcome and the evidence on treatment selection were examined as secondary objectives.

## 2. Materials and Methods

### 2.1. Protocol and Registration

This review was reported in accordance with the Preferred Reporting Items for Systematic reviews and Meta-Analyses (PRISMA) 2020 guidelines. The protocol was registered retrospectively on the Open Science Framework (OSF; DOI: 10.17605/OSF.IO/42UNZ) on 20 August 2026, after the literature search (last searched on 8 July 2026) and study screening had been completed; registration was therefore not prospective. The project was initiated as an internal review without a pre-registered protocol, and formal registration was undertaken subsequently to provide a public, time-stamped record of the eligibility criteria, outcomes, and analysis plan. Because registration followed screening, analytic decisions made after data inspection are explicitly identified as post hoc in this report: the exclusion of a randomized-trial cohort overlapping with a GCIG individual-patient-data dataset from the primary overall survival synthesis and, during revision, a re-audit of every selected effect estimate against the primary publications, with explicit rules for estimate selection, overlapping cohorts, and the cytoreduction outcome definition (see Section 2.6 and Section 2.8.9); these rules replaced an earlier post hoc exclusion of cytoreduction studies with extreme odds ratios. The corresponding sensitivity estimates are reported alongside the primary results.

### 2.2. Ethical Considerations

This study is a systematic review and meta-analysis of previously published aggregate-level data. It did not involve any collection of new data from human participants or animals by the authors, and no identifiable IPD was accessed. Thus, institutional review board approval and informed consent were not required. All included studies were conducted in accordance with the Declaration of Helsinki and obtained the relevant ethical approvals, as reported in their original publications.

### 2.3. Search Strategy and Information Sources

PubMed, EMBASE, and Cochrane Central Register of Controlled Trials (CENTRAL) databases were searched for studies reporting KELIM and/or conventional CA-125 kinetic parameters in patients with EOC from database inception up to 8 July 2026, with no language restrictions during the search stage. The full PubMed search string is presented in the Supplementary Material. All three databases were last accessed on 8 July 2026, and the search was not updated thereafter. Equivalent search strategies adapted using controlled vocabulary specific to each database (MeSH for PubMed/CENTRAL, Emtree for EMBASE) were applied to EMBASE and CENTRAL. Additional eligible studies not captured by the primary search were identified by hand-searching the reference lists of all included studies and relevant reviews and by performing forward citation chaining for all included studies through Google Scholar. This supplementary searching was applied uniformly to every included study, irrespective of its biomarker type or the direction of its findings.

### 2.4. Eligibility Criteria

Studies were required to meet all of the following eligibility criteria for inclusion: (i) enrolled patients with EOC who were treated with platinum-based chemotherapy; (ii) reported the KELIM and/or a conventional CA-125 kinetic parameter (e.g., half-life, nadir, percentage decline, exponential slope); (iii) reported at least one outcome of interest (i.e., progression-free survival [PFS], overall survival [OS], or complete cytoreduction [no macroscopic residual disease]), expressed as a hazard ratio (HR), odds ratio (OR), or discriminatory statistic (area under the curve [AUC], C-index); (iv) enrolled ≥20 patients; and (v) were published as full-text English-language articles. The exclusion criteria were as follows: (i) conference abstracts without extractable full data on the outcomes; (ii) studies restricted to recurrent or second-line treatment populations; (iii) studies that did not report a CA-125 kinetic biomarker (e.g., those reporting only a single static CA-125 value); and (iv) reviews, systematic reviews, or meta-analyses (retained only for reference-list screening, not for data extraction).

### 2.5. Study Selection

**Figure 1:**
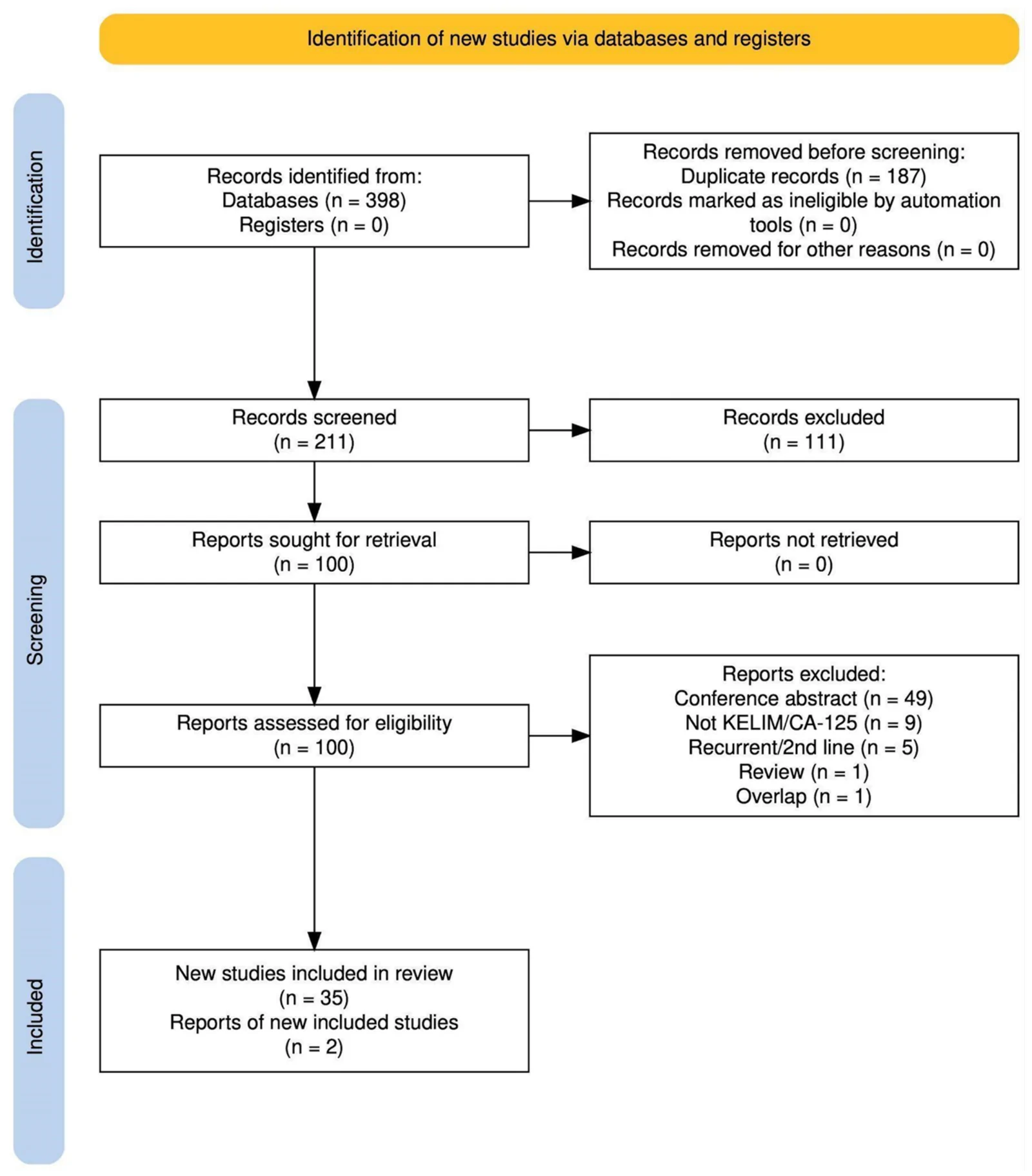
PRISMA 2020 flow diagram [44].

Records identified through the database search were exported to Rayyan for deduplication and screening. Two reviewers independently screened the titles and abstracts against the eligibility criteria using Rayyan and subsequently reviewed the full texts of all potentially eligible reports. Discrepancies during either stage were resolved through discussion between the two reviewers until consensus was reached. Reasons for full-text exclusion were recorded categorically (conference abstract without full data, biomarker not reported, recurrent/second-line treatment populations, review/meta-analysis design, or duplicate/overlapping report) to populate the PRISMA flow diagram (Figure 1). Records identified through hand-searching and citation chaining were screened in the same Rayyan project by the same two reviewers, independently, against the same eligibility criteria and with the same discrepancy-resolution procedure as the database records. This process identified two additional studies that evaluated conventional CA-125 kinetic parameters but were not captured by the formal database search. To limit the potential for selection bias, neither study contributed to any pooled estimate; both were synthesized narratively as conventional comparators, and their results were reported irrespective of their direction.

### 2.6. Data Extraction and Analysis

Two reviewers independently extracted the data using a pre-piloted standardized form. Discrepancies were resolved through discussion until consensus was reached. The following variables were extracted:

- *Study characteristics:* first author, publication year, country, study design (randomized trial sub-analysis, GCIG-IPD meta-analysis, or retrospective/real-world cohort), sample size, treatment setting (PCS vs. NACT), and follow-up duration.
- *Biomarker definition:* KELIM calculation method and standardization approach, specific conventional CA-125 kinetic parameter used (half-life, nadir, exponential slope, longitudinal model, or GCIG CA-125 response criterion), and cut-off applied to dichotomize favorable versus unfavorable kinetics.
- Outcome data: HRs for OS and PFS and ORs for complete cytoreduction comparing favorable with unfavorable KELIM (or the corresponding conventional parameter) and discriminatory statistics (AUC, C-index) if reported. Within each study, one estimate per outcome and cohort was selected using the following hierarchy, which was defined during revision and applied to all studies: (i) the dichotomous comparison at the standardized KELIM cut-off of 1.0 or at the study-specific cut-off when 1.0 was not used; when only categorical estimates were reported, the highest versus lowest category (tercile) was used and flagged; (ii) the multivariable estimate when reported, otherwise the univariable estimate (flagged); and (iii) for complete cytoreduction, when no OR was reported, an unadjusted OR calculated from published counts. Estimates for KELIM analyzed only as a continuous variable or only within composite KELIM-and-surgery groups were not pooled. The selected estimate, its location in the source publication, and the reason for any non-pooling are listed for every included study in Supplementary Table S1.
- *Treatment-interaction data*: For studies reporting biomarker-by-treatment interaction analyses (e.g., bevacizumab, dose-dense chemotherapy, atezolizumab, hyperthermic intraperitoneal chemotherapy [HIPEC]), stratum-specific HRs and interaction *p*-values were extracted separately from primary prognostic estimates and analyzed narratively rather than pooled quantitatively. This evidence was classified as (i) predictive evidence from a comparison between treatments (or treated versus untreated patients) within KELIM strata with a formal KELIM-by-treatment interaction test; (ii) composite-subgroup evidence, in which treatment benefit was shown only in patients with unfavorable KELIM combined with a second adverse factor; or (iii) prognostic evidence, in which outcomes were compared by KELIM within a single treated population. Only category i was regarded as evidence of differential treatment benefit; better outcomes within a KELIM-defined subgroup without a treatment comparison and an interaction test (categories ii and iii) were regarded as prognostic or exploratory. Because all category (i) analyses identified were post hoc or observational and none had been replicated, they were also classified as exploratory.
- *BRCA-stratified data*: when reported, KELIM values and outcome estimates stratified by BRCA mutation status were extracted separately.

For pooling purposes, studies contributing more than one independent cohort (e.g., separate trial populations or separate treatment-setting cohorts reported within a single publication) were treated as separate data rows. When two publications reported on the same or largely the same patients (the same trial or the same institution with overlapping accrual periods), only the larger cohort was included in the primary analysis and the other was examined in a sensitivity analysis; when overlap was possible but limited (e.g., one institution contributing to a multicenter registry), both were included and the smaller cohort was removed in a sensitivity analysis. Because the syntheses addressed first-line treatment of predominantly advanced (stage III–IV) disease, the GCIG-IPD analysis restricted to stage I–II disease [9] was not pooled with the advanced-stage cohorts and is reported separately.

### 2.7. Risk-of-Bias Assessment

Two reviewers independently evaluated the risk of bias for each included study using a design-appropriate tool. Study participation, study attrition, prognostic factor measurement (KELIM/CA-125 kinetic ascertainment), outcome measurement, study confounding, and statistical analysis and reporting were assessed using the Quality in Prognosis Studies (QUIPS) tool [10]. The risk of bias with respect to these six domains was rated as low, moderate, or high. An overall risk-of-bias judgment was assigned to each study based on the pattern of domain-level ratings. Disagreements were resolved through discussion between the two reviewers until consensus was reached, the same as for study selection and data extraction.

### 2.8. Statistical Analysis

#### 2.8.1. Pooling Method

Effect estimates were pooled using the generic inverse-variance method, with the natural log of each reported HR/OR and its standard error (SE = [ln(upper CI) − ln(lower CI)]/3.92) derived from the 95% confidence interval (CI).

#### 2.8.2. Effect Model

A random-effects model with the DerSimonian–Laird estimator was applied to all pooled analyses a priori, anticipating clinical and methodological heterogeneity across study designs, biomarker cut-offs, and treatment settings.

#### 2.8.3. Orientation Standardization

All effect estimates were oriented to a common reference direction (favorable KELIM/CA-125 kinetics as the index group, unfavorable as the comparator), with HRs/ORs and corresponding CI bounds being inverted when studies reported the inverse relationship.

#### 2.8.4. Outcome-Specific Pooling

Given their distinct clinical and mechanistic nature, OS, PFS, and complete cytoreduction were examined as separate outcomes in independent pooled models, each generating its own summary estimate, 95% CI, I^2^, and significance test. Conventional CA-125 kinetic parameters were pooled separately from KELIM, and the two sets of pooled estimates are presented descriptively; no formal statistical comparison between the biomarker classes was performed. Because the estimates derive from different populations, similar HRs or overlapping CIs were not interpreted as evidence of equivalence or superiority. Throughout this review, “prognostic” denotes an association between a biomarker and outcome (HR or OR), “discrimination” denotes the ability to separate patients with and without an outcome (AUC or C-index), and “predictive” is reserved for modification of treatment benefit by the biomarker (a biomarker-by-treatment interaction). HRs and ORs quantify the strength of association and were not used to compare discriminatory performance, which was compared only using measures of the same type (AUC or C-index) and directly only where both biomarkers had been evaluated in the same cohort.

#### 2.8.5. Assessment of Trial-Level Overlap

Because several included studies derive from overlapping trial networks, the trial lists of the individual-patient-data (IPD) meta-analyses contributing pooled KELIM estimates were cross-checked against the randomized trials reported separately as trial-level sub-analyses. Of the three trials reported separately by Colomban et al. [4], ICON-7 is one of the eight first-line randomized trials whose individual patient data underlie the Corbaux et al. [6] GCIG-IPD (MAOV) estimate, whereas AGO-OVAR-9 and AGO-OVAR-7 do not. To avoid double-counting, the ICON-7 estimate was excluded from the primary pooled OS analysis, and the AGO-OVAR-9 and AGO-OVAR-7 estimates were included; an estimate including ICON-7 is reported as a sensitivity analysis. In the originally submitted analysis, AGO-OVAR-9 had also been excluded on the incorrect assumption that it contributed to the GCIG-IPD dataset, and AGO-OVAR-7 had not been extracted; both were corrected on re-audit.

#### 2.8.6. Cytoreduction Outcome Definition

Regarding complete cytoreduction, eligibility for pooling was determined by the outcome definition rather than by the size of the estimate: studies were pooled if they reported, or allowed calculation of, an OR for complete cytoreduction (no macroscopic residual disease: R0, CC-0, or no gross residual disease) by dichotomized KELIM. Studies reporting only optimal cytoreduction (residual disease < 1 cm) or a combined R0/R1 outcome were not pooled, and no study was excluded because of the magnitude of its estimate. This rule replaced the post hoc exclusion, in the originally submitted analysis, of two studies with extreme ORs (>30); under it, Zouzoulas et al. [11] (complete cytoreduction; OR calculated from published counts) is pooled, whereas Wang et al. [12] (satisfactory [R0/R1] cytoreduction) is not. The re-audit also identified two additional cohorts with extractable counts for complete cytoreduction [13,14] and corrected two estimates that did not match their sources [5,15]. Sensitivity analyses excluded Zouzoulas et al. [11], whose estimate rests on a single incomplete resection in the favorable-KELIM group, applied a Haldane–Anscombe continuity correction (0.5 added to each cell) to its counts, and added Wang et al. [12] using the inverse of its published multivariable OR.

#### 2.8.7. Heterogeneity

Between-study heterogeneity was quantified using *I*^2^ and *τ*^2^ (between-study variance). *I*^2^ was interpreted as follows: 25%, low; 50%, moderate; and 75%, substantial. Cochran’s Q test was performed to examine whether the observed heterogeneity exceeded that expected by chance. Ninety-five percent prediction intervals were additionally calculated post hoc for the KELIM syntheses (t distribution with k − 2 degrees of freedom) to indicate the range of effects expected in a new comparable cohort. Substantial heterogeneity was addressed by (i) applying a random-effects model to all syntheses; (ii) a prespecified subgroup analysis by study design; (iii) post hoc subgroup analyses and univariable meta-regression by KELIM contrast, treatment setting, and treatment era; (iv) sensitivity and leave-one-out influence analyses (described below); and (v) interpreting pooled estimates as average effects across heterogeneous cohorts rather than as precise effect sizes.

#### 2.8.8. Subgroup Analysis

Prespecified subgroup analysis was conducted for OS-stratified studies according to design: (i) randomized trial sub-analyses and GCIG-IPD meta-analysis versus (ii) retrospective/real-world cohorts. Separate pooled HRs were generated for each stratum. For subgroup differences (df = k − 1), a formal chi-square test was performed to examine whether the study design significantly modified the pooled effect.

Additional post hoc exploratory analyses of the primary OS synthesis were performed to investigate residual heterogeneity: subgroup analyses and univariable random-effects meta-regression (method-of-moments estimator of *τ*^2^) according to (i) KELIM contrast (dichotomous at the standardized 1.0 cut-off, dichotomous at another cut-off, or highest vs. lowest tercile), (ii) treatment setting (PCS vs. NACT), and (iii) treatment era (accrual completed before the introduction of first-line PARP inhibitor maintenance vs. accrual extending into the PARP inhibitor era), together with a leave-one-out influence analysis. Study-level classifications are given in Supplementary Table S2. Given the small number of cohorts, multivariable meta-regression was not performed.

#### 2.8.9. Sensitivity Analyses

The following sensitivity analyses were performed. (i) Cohort overlap: for OS, the primary analysis was repeated including the ICON-7 cohort (15 cohorts); for OS and PFS, the Zouzoulas et al. [11] cohort was replaced by the overlapping Theodoulidis et al. cohort from the same institution, and cohorts with possible partial overlap were removed (Bouvarel et al. [15], from one center that contributes to the registry analyzed by Becker et al.; and the interval-surgery cohort of Kim et al., which may share patients with Cho et al.). (ii) Estimate type: cohorts contributing highest-versus-lowest tercile rather than dichotomous estimates were removed. (iii) Cytoreduction: Zouzoulas et al. [11] was removed or continuity-corrected, and Wang et al. [12] was added, as described above. (iv) Leave-one-out analysis: each random-effects model was re-fitted omitting one cohort at a time, and the pooled estimate, 95% CI, I^2^, and *τ*^2^ were recorded to identify cohorts with disproportionate influence on the pooled estimate or on heterogeneity. For subgroup analyses, a separate random-effects estimate was obtained within each subgroup, and differences between subgroups were tested using the between-subgroup Q statistic (chi-square test with degrees of freedom equal to the number of subgroups minus one).

#### 2.8.10. Certainty of Evidence and Small-Study Effects

The certainty of evidence for each pooled outcome, and for the indirect comparison of KELIM with CA-125 half-life, was rated post hoc using the Grading of Recommendations Assessment, Development and Evaluation (GRADE) approach for prognostic factor research [16]. Because the question concerned the association between a prognostic factor and outcomes in cohorts (including post hoc analyses of randomized trials, analyzed as cohorts), evidence started at high certainty and was downgraded for risk of bias, inconsistency (judged from the direction of individual estimates, I^2^, and the 95% prediction interval), indirectness, imprecision, and publication bias. Small-study effects were assessed for syntheses including at least 10 cohorts by visual inspection of funnel plots and Egger’s regression test [17].

#### 2.8.11. Narrative Synthesis

Narrative synthesis was performed for data that could not be quantitatively pooled, including treatment-selection/biomarker-by-treatment interaction analyses, discriminatory statistic comparisons (AUC, C-index) between KELIM and conventional CA-125 parameters, HIPEC-stratified outcomes (due to the conflicting direction of effect across the two available studies), and BRCA-stratified prognostic groupings.

#### 2.8.12. Software

Pooled, subgroup, meta-regression, and sensitivity analyses were performed with the DerSimonian–Laird random-effects method implemented in Python (version 3.9.6; NumPy [version 2.0.2] and SciPy [version 1.13.1]), and forest and funnel plots were generated with Matplotlib (version 3.9.4). Statistical significance was set at a two-sided *p*-value of <0.05 for all pooled and subgroup analyses.

## 3. Results

### 3.1. Study Selection and Characteristics

A total of 398 records (89 in PubMed, 72 in CENTRAL, and 237 in EMBASE) were identified through the formal search. Following the removal of 187 duplicates, title and abstract screening yielded 211 records, among which 111 were excluded. The full texts of 100 remaining reports were assessed, leading to the exclusion of 65 records (49 conference abstracts, 9 studies not reporting a KELIM/CA-125 kinetic biomarker, 5 studies restricted to recurrent/second-line treatment populations, 1 systematic review, and 1 overlapping report; Supplementary Table S3) and to the identification of 35 studies that met the inclusion criteria. Two additional conventional CA-125 kinetic comparator studies [18,19] were identified through hand-searching and citation chaining, resulting in the inclusion of 37 studies. Among these 37 included studies, 29 evaluated KELIM or an equivalent modeled CA-125 approach, whereas 8 assessed conventional (non-modeled) CA-125 kinetic parameters (Table 1 and Figure 1); this includes Ducoulombier et al. [20], which modeled CA-125 kinetics using a KELIM-equivalent approach prior to the KELIM nomenclature and was accordingly counted among the KELIM cohorts. The KELIM studies comprised randomized trial sub-analyses [2,4,5,21], GCIG IPD meta-analyses [6,9], and multiple retrospective/real-world cohorts spanning Europe, Asia, and North America, including the first large national-registry validation (Netherlands, *n* = 1582) [7] and the first PARP inhibitor era cohort [22].

**Table 1:** Characteristics of the 37 included studies.

Study	Year	Country	Design	*n*	Setting	Biomarker	Cut-off	Key Outcomes	RoB
RCT sub-analyses/GCIG-IPD meta-analyses									
You et al. [5]	2020	France	Phase II RCT sub-analysis (CHIVA)	134	NACT	KELIM	≥1.0(0.07/d); tercile for survival	Cytoreduction, PtRR, PFS, OS	Low
You et al. [2]	2022	USA	Phase III RCT sub-analysis (GOG-0218)	1662	First-line	KELIM	≥1.0	Bevacizumab benefit, PFS, OS	Low
Colomban et al. [4]	2019	Multinational	Phase III RCT sub-analysis (AGO OVAR 9/7, ICON-7)	2868	First-line	KELIM	Tercile	PFS, OS, vs. GCIG criterion	Low
Colomban et al. [23]	2023	UK	Phase III RCT sub-analysis (ICON-8)	1334	First-line	KELIM	≥1.0	Dose-dense benefit, PFS, OS	Low
Corbaux et al. [6]	2023	Multinational	GCIG-IPD meta-analysis (8 RCTs)	5884	First-line	KELIM	≥1.0	PFS, OS	Low
Corbaux et al. [9]	2026	Multinational	GCIG-IPD meta-analysis (early stage)	1143	Adjuvant	KELIM	≥1.0	RFS, OS (stage I–II)	Low
You et al. [21]	2025	Multinational	Phase III RCT sub-analysis (IMagyn050)	1199	First-line	KELIM	≥1.0	Atezolizumab benefit, PFS	Low
Cho et al. [24]	2026	Korea	Pooled (KGOG3042 + KOV-HIPEC-01)	213	NACT	KELIM	≥1.0	HIPEC benefit, PFS, OS	Low
KELIM retrospective/real-world cohorts									
van Wagensveld et al. [7]	2024	Netherlands	National registry	1582	NACT	KELIM	≥1.0	Cytoreduction, PFS, OS	Moderate
Becker et al. [25]	2025	France	Nationwide registry	743	NACT	KELIM + BRCA	≥1.0	PFS, OS	Moderate
Bouvarel et al. [15]	2024	France	Retrospective cohort	284	NACT	KELIM	≥1.0	Cytoreduction, PtRR, PFS, OS	Moderate
Pham et al. [26]	2026	Vietnam	Retrospective (nomogram)	418	Mostly PCS (90%)	KELIM	≥1.0	OS	Moderate
Marchetti et al. [27]	2025	Italy	Retrospective cohort	373	NACT + PARPi	KELIM + CRS + HRD	Continuous	PARPi PFS	Moderate
Sinukumar et al. [28]	2026	India	Multi-institution, PS-matched	265	NACT	KELIM	≥1.0	HIPEC, OS, DFS	Moderate
Kim et al. [22]	2024	Korea	Multicenter retrospective	252	First-line + PARPi	KELIM	≥1.0	PFS, cytoreduction	Moderate
Piedimonte et al. [29]	2022	Canada	Retrospective cohort	217	NACT	KELIM	≥1.0	Cytoreduction, PFS, OS	Moderate
Piedimonte et al. [30]	2024	Canada	Retrospective cohort	172	NACT	KELIM + CRS	≥1.0	PFS (KELIM × CRS)	Moderate
Oufkir et al. [31]	2024	France	Retrospective (Institut Curie)	149	NACT	Std KELIM + PtRR	≥1.0	Platinum sensitivity, OS	Moderate
Li et al. [32]	2024	China	Retrospective (2 centers)	133	NACT	KELIM	≥0.925	Cytoreduction, PFS, OS	Moderate
Liu et al. [33]	2025	China	Retrospective cohort	114	First-line	KELIM + LDH	≥1.0	Bevacizumab benefit, PFS	Moderate
Khaled et al. [13]	2025	Belgium	Retrospective cohort	111	Mostly NACT (86%)	KELIM	≥1.0	Cytoreduction, PFS, OS	Moderate
Yalcin et al. [14]	2025	Turkey	Retrospective cohort	102	Mixed	KELIM	≥1.0	Cytoreduction, PFS, OS	Moderate
Parić et al. [34]	2026	Bosnia	Retrospective real-world	92	Mixed	KELIM	≥1.0	PFS, OS	Moderate
Zouzoulas et al. [11]	2024	Greece	Retrospective cohort	83	NACT	KELIM	≥1.0	Cytoreduction, PFS, OS	Moderate
Theodoulidis et al. [35]	2025	Greece	Retrospective cohort	78	NACT	KELIM + NLR	≥1.0	PFS, OS	Moderate
Wang et al. [12]	2026	China	Retrospective cohort	70	NACT	KELIM	≥1.0	Cytoreduction, PFS, OS	Moderate
Matsukuma et al. [36]	2024	Japan	Retrospective cohort	55	NACT	KELIM + CRS + MDR1	Tercile	CRS association	Moderate
Lv et al. [37]	2025	China	Retrospective cohort	52	Mixed	KELIM	≥1.0	PFS	Moderate
Ducoulombier et al. [20]	2017	France	Retrospective cohort	54	NACT	Modeled CA-125 (KELIM)	Model	Cytoreduction	Moderate
Conventional CA-125 studies									
Riedinger et al. [38]	2006	France	Multicenter retrospective	631	First-line	CA-125 half-life, nadir	14 d; 20 kU/L	DFS, OS	Moderate
Chiang et al. [39]	2014	Taiwan	Retrospective cohort	275	First-line	Longitudinal CA-125	Model	OS prediction	Moderate
Gadducci et al. [40]	1995	Italy	Multicentric retrospective	225	First-line	CA-125 half-life	14 d	PCR, OS	Moderate
Buller et al. [41]	1996	USA	Retrospective cohort	126	First-line	CA-125 slope	7.6 d	OS	Moderate
Gadducci et al. [42]	2004	Italy	Retrospective cohort	71	First-line	CA-125 half-life	14 d	CR, PFS, OS	Moderate
Markowska et al. [43]	1992	Poland	Prospective cohort	130	First-line	CA-125 half-life	—	Survival	Moderate
Karamouza et al. [19]	2023	Multinational	GCIG-IPD meta-analysis (CA-125 dynamics)	5573	First-line	Conventional CA-125 dynamics (ORD, baseline, 3-mo value)	Model	OS discrimination	Low
Lee et al. [18]	2018	Korea	Retrospective cohort	210	NACT	Ten conventional CA-125 kinetic models	Model	Cytoreduction, OS/PFS discrimination	Moderate

CA-125, cancer antigen-125; CR, complete response; CRS, chemotherapy response score; DFS, disease-free survival; GCIG, Gynecologic Cancer InterGroup; HIPEC, hyperthermic intraperitoneal chemotherapy; HRD, homologous recombination deficiency; IPD, individual patient data; KELIM, CA-125 elimination rate constant K; kU/L, kilounits per liter; LDH, lactate dehydrogenase; MDR1, multidrug resistance protein 1; NACT, neoadjuvant chemotherapy; NLR, neutrophil-to-lymphocyte ratio; ORD, overall rate of decline; OS, overall survival; PARPi, poly(ADP-ribose) polymerase inhibitor; PCR, pathological complete response; PCS, primary cytoreductive surgery; PFS, progression-free survival; PS-matched, propensity score-matched; PtRR, platinum-resistant relapse; RCT, randomized controlled trial; RFS, recurrence-free survival; RoB, risk of bias; Std KELIM, standardized KELIM.

### 3.2. KELIM and OS

**Figure 2:**
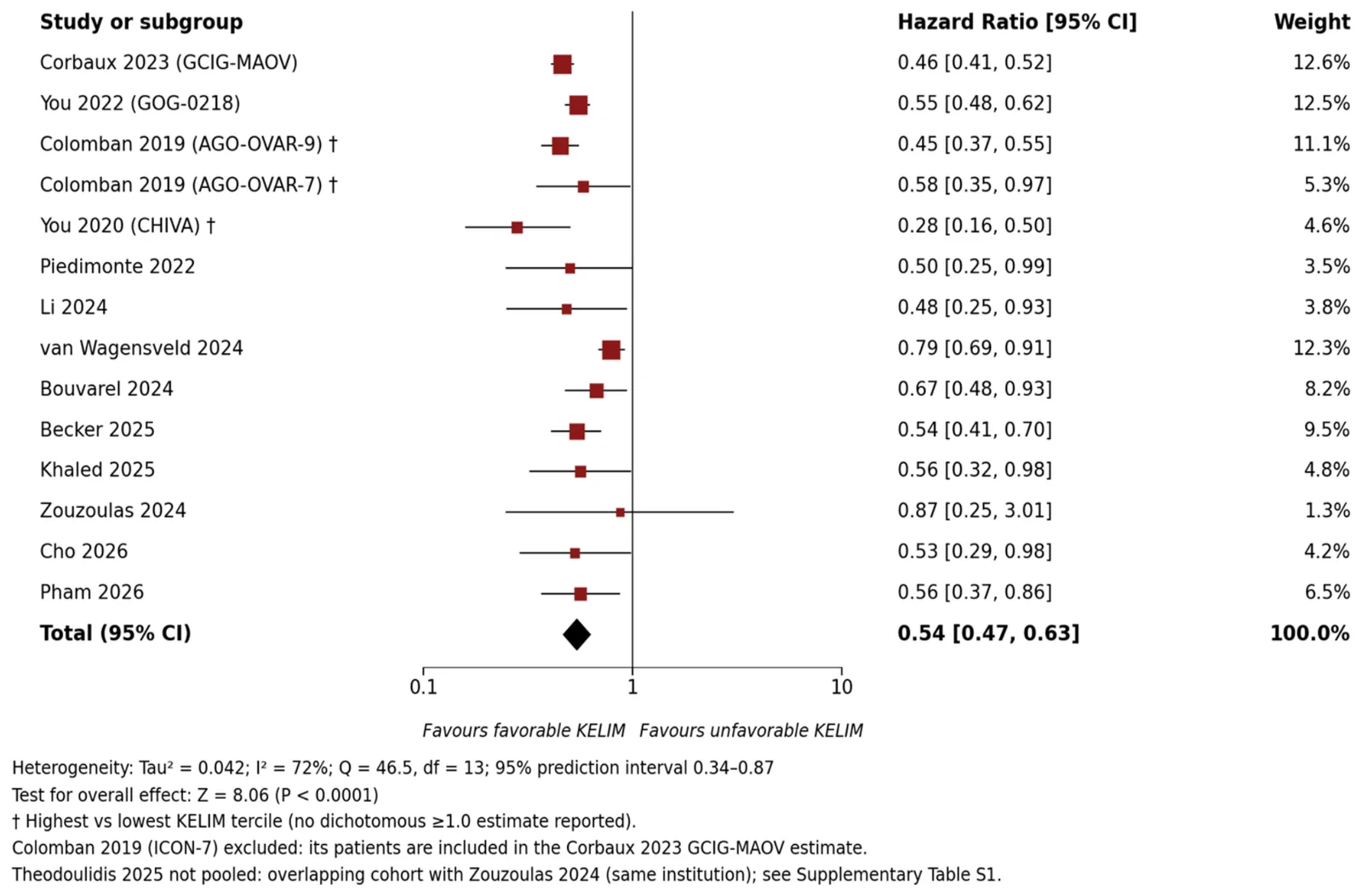
Forest plot of the KELIM score and overall survival, corrected for cohort overlap (14 independent cohorts). Pooled HR: 0.54 (95% CI: 0.47–0.63, I^2^ = 72%). Included cohorts: Corbaux 2023 (GCIG-MAOV) [6], You 2022 (GOG-0218) [2], Colomban 2019 (AGO-OVAR-9 and AGO-OVAR-7) [4], You 2020 (CHIVA) [5], Piedimonte 2022 [29], Li 2024 [32], van Wagensveld 2024 [7], Bouvarel 2024 [15], Becker 2025 [25], Khaled 2025 [13], Zouzoulas 2024 [11], Cho 2026 [24], and Pham 2026 [26]. Colomban 2019 (ICON-7) [4] and Theodoulidis 2025 [35] were not pooled because of cohort overlap.

HRs for OS comparing favorable with unfavorable KELIM were extractable for 16 cohorts. After excluding the ICON-7 cohort, whose patients are included in the Corbaux et al. [6] GCIG-IPD (MAOV) estimate, and the Theodoulidis et al. [35] cohort, which overlaps with Zouzoulas et al. [11] (see Section 2), the primary analysis pooled 14 independent cohorts (approximately 12,900 patients): favorable KELIM was associated with significantly improved OS (pooled HR: 0.54, 95% CI: 0.47–0.63, I^2^ = 72%, Z = 8.06, *p* < 0.0001; Figure 2). The 95% prediction interval was 0.34–0.87 (Cochran’s Q = 46.5, df = 13, *p* < 0.001), indicating that favorable KELIM would still be expected to be associated with improved OS in a new comparable cohort despite this heterogeneity. Effect estimates were directionally consistent across all cohorts, with HRs ranging from 0.28 [5] to 0.87 [11]; three trial cohorts contributed highest-versus-lowest tercile rather than dichotomous estimates [4,5]. The pooled estimate was materially unchanged when ICON-7 was included (HR: 0.53, 95% CI: 0.46–0.61; 15 cohorts; Supplementary Figure S1), when Theodoulidis et al. [35] replaced Zouzoulas et al. [11] (HR: 0.54, 95% CI: 0.46–0.62), when Bouvarel et al. [15] was removed (HR: 0.53, 95% CI: 0.45–0.62), and when the tercile-based estimates were removed (HR: 0.57, 95% CI: 0.49–0.68; 11 cohorts) (Supplementary Table S2).

### 3.3. Subgroup Analysis by Study Design

**Figure 3:**
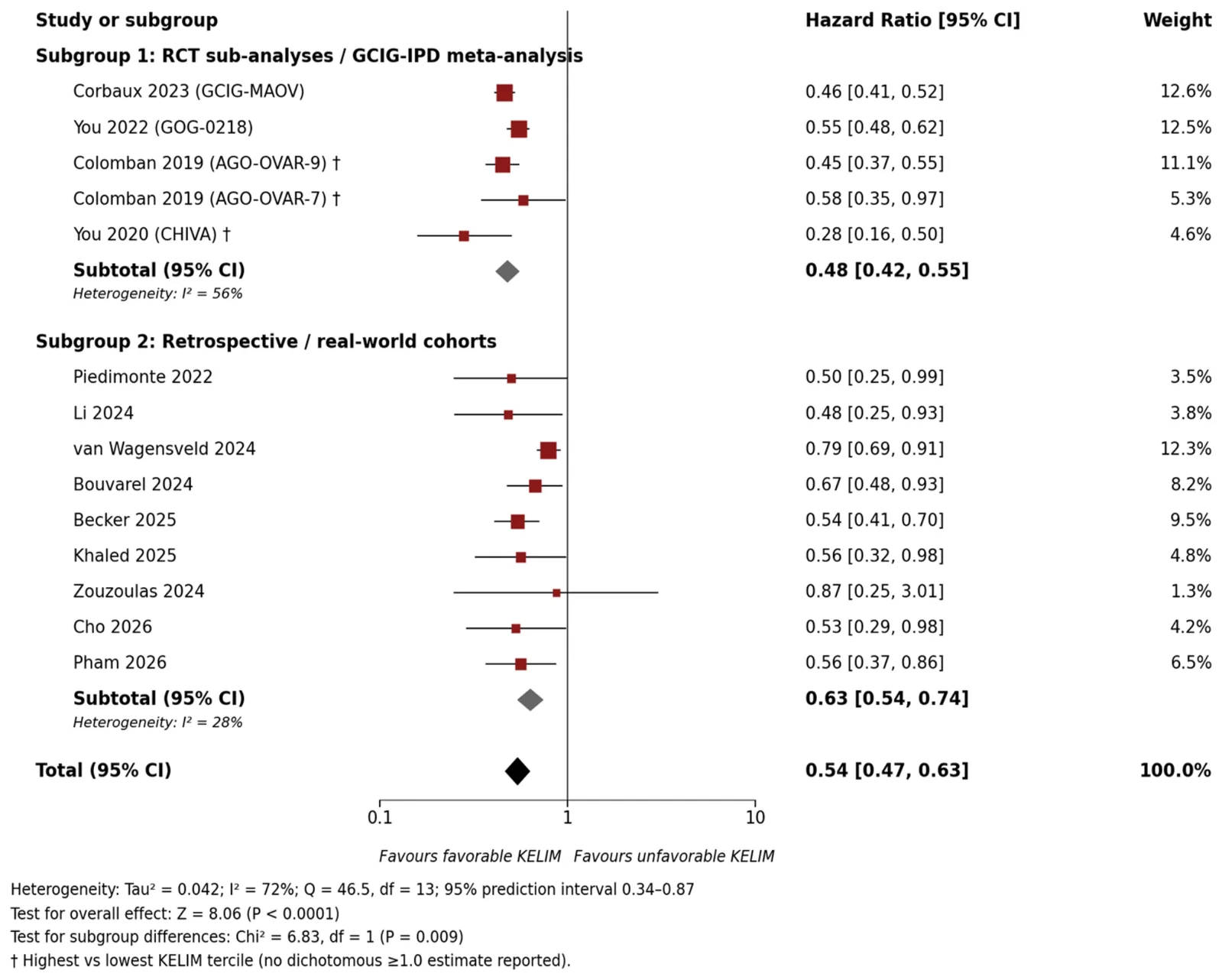
Subgroup analysis of the KELIM score and overall survival by study design: randomized trial/GCIG-IPD (HR: 0.48, 5 cohorts) versus retrospective/real-world (HR: 0.63, 9 cohorts). Test for subgroup difference *p* = 0.009. Randomized trial/GCIG-IPD subgroup: Corbaux 2023 (GCIG-MAOV) [6], You 2022 (GOG-0218) [2], Colomban 2019 (AGO-OVAR-9 and AGO-OVAR-7) [4], and You 2020 (CHIVA) [5]. Retrospective/real-world subgroup: Piedimonte 2022 [29], Li 2024 [32], van Wagensveld 2024 [7], Bouvarel 2024 [15], Becker 2025 [25], Khaled 2025 [13], Zouzoulas 2024 [11], Cho 2026 [24], and Pham 2026 [26].

Heterogeneity was partly attributable to study design (Figure 3). Pooled HRs for OS were 0.48 (95% CI: 0.42–0.55, I^2^ = 56%; five cohorts) in randomized trial sub-analyses/GCIG-IPD meta-analysis and 0.63 (95% CI: 0.54–0.74, I^2^ = 28%; nine cohorts) in retrospective/real-world cohorts (test for subgroup differences: chi-square = 6.83, df = 1, *p* = 0.009). KELIM remained a significant prognostic factor in both settings, but the association was weaker in real-world cohorts, in which patient selection, chemotherapy regimens, and CA-125 sampling are less standardized.

### 3.4. Exploration of Residual Heterogeneity

In post hoc exploratory analyses (Supplementary Table S2), neither KELIM contrast, treatment setting, nor treatment era significantly modified the association between KELIM and OS. Pooled HRs were 0.58 (95% CI: 0.49–0.69, I^2^ = 75%; 10 cohorts) for dichotomous estimates at the standardized 1.0 cut-off, 0.48 (95% CI: 0.25–0.93; one cohort) for a dichotomous estimate at another cut-off, and 0.43 (95% CI: 0.32–0.59, I^2^ = 44%; three cohorts) for highest-versus-lowest tercile estimates (P for subgroup difference = 0.260). Pooled HRs were 0.50 (95% CI: 0.45–0.55; five cohorts) after PCS and 0.57 (95% CI: 0.46–0.71; nine cohorts) after NACT (*p* = 0.265) and 0.54 (95% CI: 0.45–0.64; nine cohorts) for cohorts accrued entirely before the PARP inhibitor era versus 0.54 (95% CI: 0.40–0.73; five cohorts) for cohorts whose accrual extended into it (*p* = 0.982). In the leave-one-out analysis, the pooled HR ranged from 0.51 to 0.56; omitting the Netherlands registry [7] alone reduced I^2^ from 72% to 14% (HR: 0.51, 95% CI: 0.46–0.55), whereas omitting any other cohort left I^2^ between 65% and 74%. The residual heterogeneity therefore arose mainly from this single cohort, the largest real-world registry, whose patients were diagnosed in 2008–2011, received heterogeneous chemotherapy regimens, and showed one of the weakest associations (HR: 0.79), together with the difference between trial-based and real-world cohorts, rather than from a systematic effect of cut-off, treatment setting, or era. With fourteen cohorts and only one to five cohorts in several subgroups, these analyses had limited power to detect effect modification.

### 3.5. KELIM and PFS

**Figure 4:**
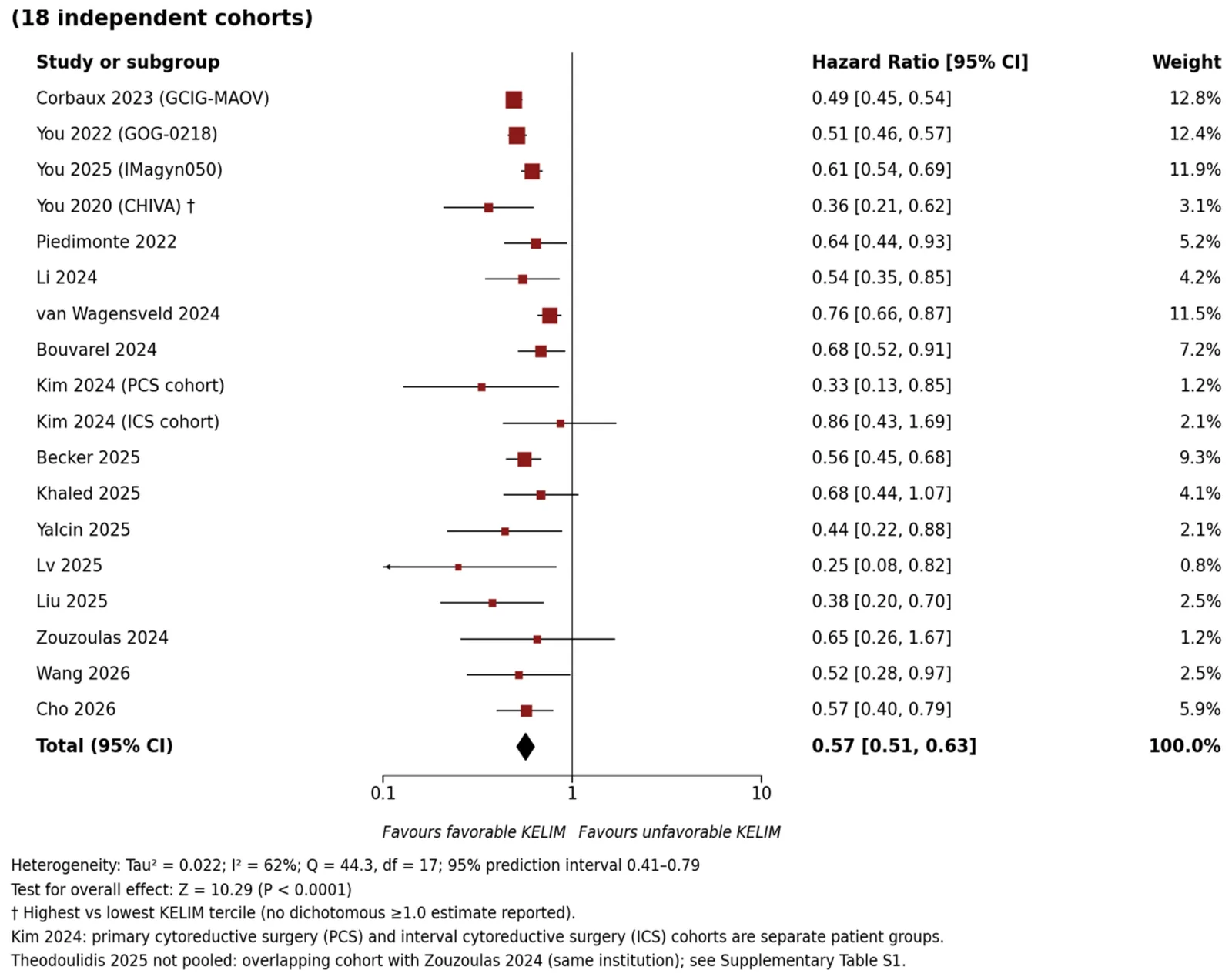
Forest plot of the KELIM score and progression-free survival (18 independent cohorts; pooled HR = 0.57, 95% CI: 0.51–0.63, I^2^ = 62%). Included cohorts: Corbaux 2023 (GCIG-MAOV) [6], You 2022 (GOG-0218) [2], You 2025 (IMagyn050) [21], You 2020 (CHIVA) [5], Piedimonte 2022 [29], Li 2024 [32], van Wagensveld 2024 [7], Bouvarel 2024 [15], Kim 2024 (PCS and ICS cohorts) [22], Becker 2025 [25], Khaled 2025 [13], Yalcin 2025 [14], Lv 2025 [37], Liu 2025 [33], Zouzoulas 2024 [11], Wang 2026 [12], and Cho 2026 [24]. Theodoulidis 2025 [35] was not pooled because of cohort overlap with Zouzoulas 2024 [11].

HRs for PFS were extractable for 19 cohorts; after excluding the Theodoulidis et al. [35] cohort, which overlaps with Zouzoulas et al. [11], 18 independent cohorts (approximately 12,800 patients) were pooled. Favorable KELIM was associated with significantly improved PFS (pooled HR: 0.57, 95% CI: 0.51–0.63, I^2^ = 62%, Z = 10.29, *p* < 0.0001; Figure 4). The 95% prediction interval was 0.41–0.79 (Cochran’s Q = 44.3, df = 17, *p* < 0.001). The estimate was robust to replacing Zouzoulas et al. [11] with Theodoulidis et al. [35] (HR: 0.56, 95% CI: 0.51–0.63), to removing cohorts with possible partial overlap (HR: 0.55, 95% CI: 0.49–0.62; 16 cohorts), and to removing the tercile-based estimate [5] (HR: 0.58, 95% CI: 0.52–0.64); omitting the Netherlands registry [7] reduced I^2^ to 28% (Supplementary Table S2). In early-stage disease, analyzed separately, the GCIG-IPD analysis of 1143 patients with stage I–II disease [9] reported multivariable HRs of 0.61 (95% CI: 0.48–0.77) for recurrence-free survival and 0.50 (95% CI: 0.36–0.68) for OS with favorable KELIM, consistent in direction with the advanced-stage estimates.

### 3.6. Conventional CA-125 Parameters and Survival

**Figure 5:**
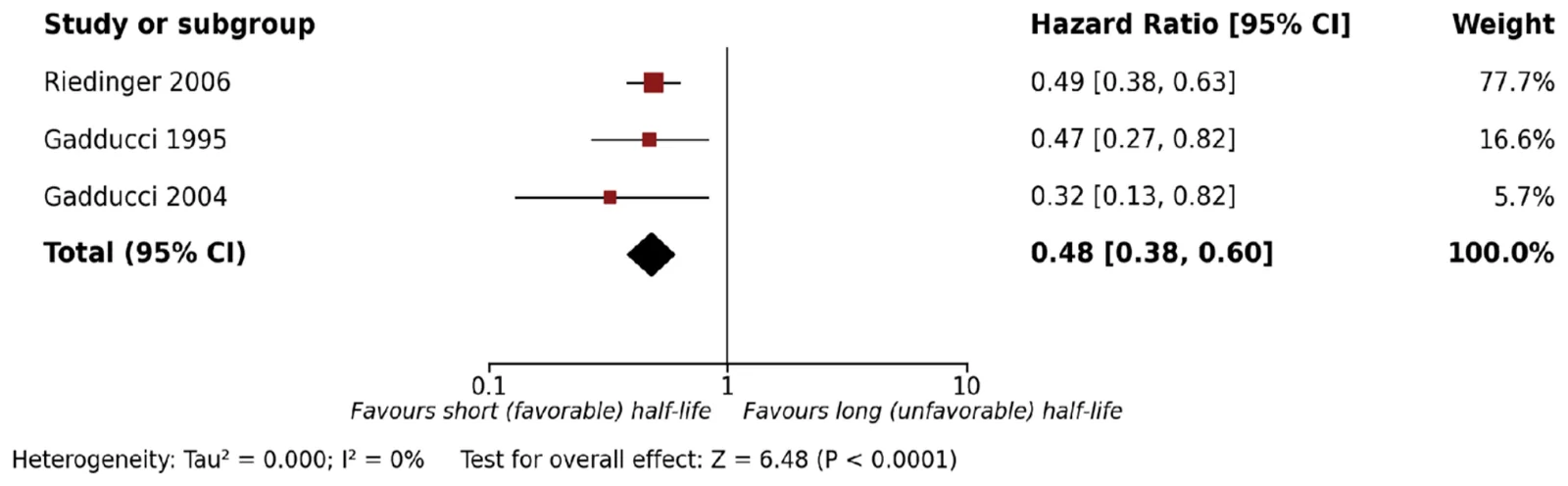
Forest plot of CA-125 half-life (short vs. long) and overall survival (3 cohorts; pooled HR = 0.48, 95% CI: 0.38–0.60, I^2^ = 0%). Included cohorts: Riedinger 2006 [38], Gadducci 1995 [40], and Gadducci 2004 [42].

Among conventional CA-125 parameters, CA-125 half-life yielded the pooled HR for OS closest in magnitude to that of KELIM. Pooling the three studies reporting adjusted HRs [38,40,42] yielded an HR of 0.48 (95% CI: 0.38–0.60, I^2^ = 0%) for OS (Figure 5). This estimate was similar in magnitude to the pooled KELIM estimate (HR: 0.54); however, because the two estimates derive from different populations and eras, and HRs measure the strength of association rather than discrimination, this similarity does not demonstrate equivalent prognostic performance. Additional studies evaluating conventional CA-125 parameters also supported the prognostic value of half-life-based approaches: the CA-125 exponential slope was the strongest independent prognostic factor for survival in one cohort [41], CA-125 half-life was independently prognostic in an Italian multicentric study [40], and a longitudinal CA-125 model had slightly higher accuracy for predicting survival than half-life/nadir (85.4% vs. 82.2%) [39]). Notably, a much larger GCIG-IPD cohort (*n* = 5573) applied a mixed-effects modeling approach to summarize each patient’s early CA-125 trajectory and cross-validated how well this summary forecasts OS; the best-performing version reached an AUC of 0.75 (95% CI: 0.70–0.80) at 2- and 3-year follow-up [19], indicating that sophisticated conventional CA-125 trajectory modeling can achieve moderate discrimination; because KELIM was not evaluated in that cohort, their relative performance cannot be determined. The only direct, within-cohort comparison involved the GCIG CA-125 response criterion: in three phase III trials, its discrimination (C-index: 0.49–0.54) was lower than that of KELIM and added no value to models already containing KELIM [4].

### 3.7. KELIM and Complete Cytoreduction

**Figure 6:**
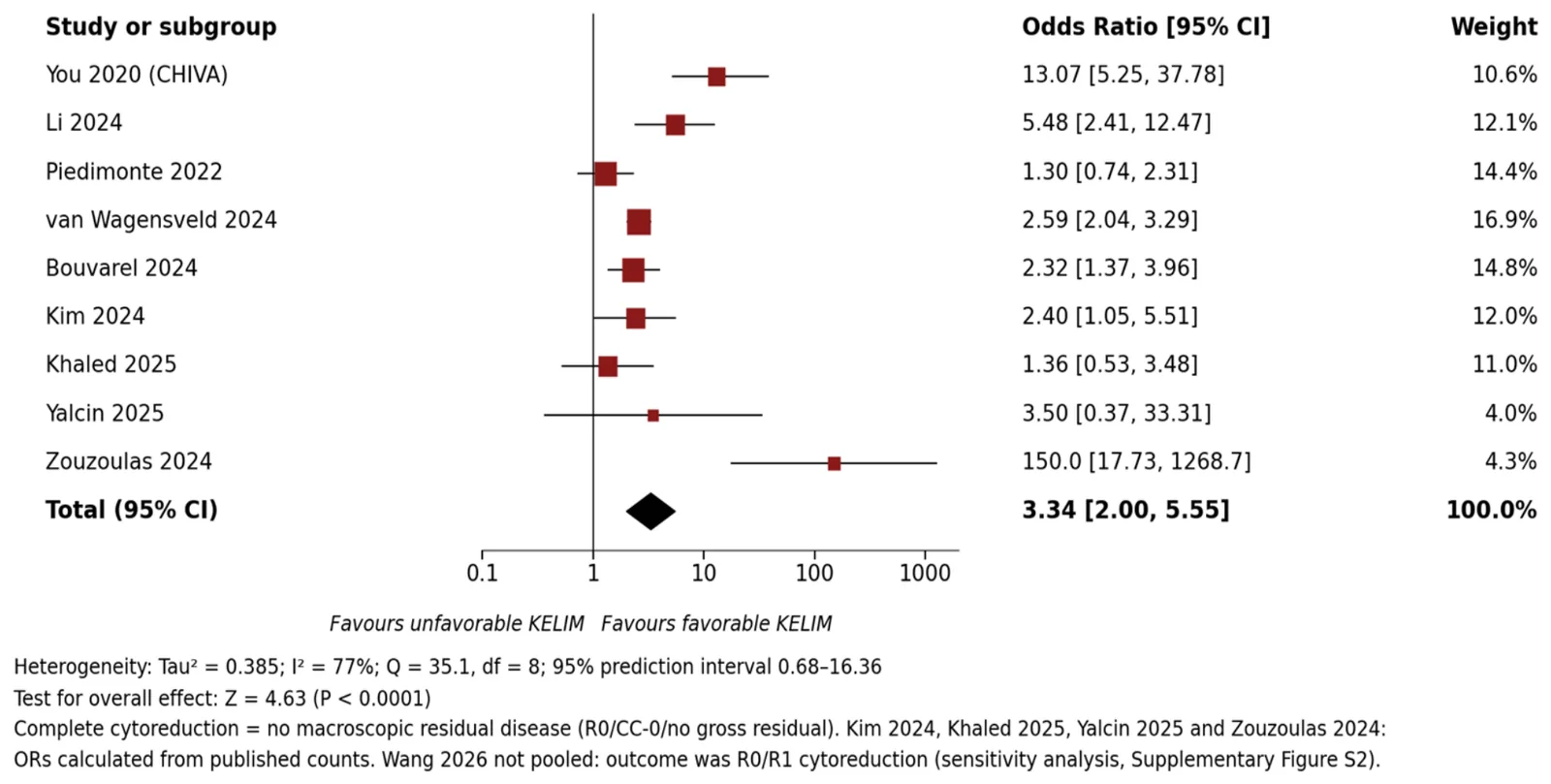
Forest plot of KELIM score and complete cytoreduction (9 cohorts). Pooled OR 3.34 (95% CI 2.00–5.55); I-squared = 77%. Included cohorts: You 2020 (CHIVA) [5], Li 2024 [32], Piedimonte 2022 [29], van Wagensveld 2024 [7], Bouvarel 2024 [15], Kim 2024 [22], Khaled 2025 [13], Yalcin 2025 [14], and Zouzoulas 2024 [11]. Wang 2026 [12] was not pooled because its outcome was R0/R1 cytoreduction.

ORs for complete cytoreduction by dichotomized KELIM were reported in, or could be calculated from published counts for, nine cohorts. Favorable KELIM was associated with significantly higher odds of complete cytoreduction (pooled OR: 3.34, 95% CI: 2.00–5.55, I^2^ = 77%, Z = 4.63, *p* < 0.0001; Figure 6). Individual estimates ranged from 1.30 (95% CI: 0.74–2.31) [29] to 150 (95% CI: 17.7–1269) [11]; the latter reflects a single incomplete resection among 51 patients with favorable KELIM. The 95% prediction interval was wide and crossed 1 (0.68–16.36; Cochran’s Q = 35.1, df = 8, *p* < 0.001), indicating that a positive association may not be observed in every setting. The estimate was robust in sensitivity analyses: excluding Zouzoulas et al. [11] gave an OR of 2.74 (95% CI: 1.80–4.16, I^2^ = 67%), applying a continuity correction to its counts gave 3.43 (95% CI: 2.05–5.74), and adding Wang et al. [12], whose outcome was satisfactory (R0/R1) cytoreduction (OR ≈ 30.3, the inverse of its reported multivariable OR of 0.033 [95% CI: 0.008–0.132]), gave 4.07 (95% CI: 2.36–7.02, I^2^ = 81%; Supplementary Figure S2); leave-one-out estimates ranged from 2.74 to 3.92. With respect to discrimination, none of the ten conventional CA-125 kinetic models achieved an AUC above 0.60 for predicting complete cytoreduction (AUC: 0.50–0.57) [18], whereas KELIM attained AUCs ranging from 0.71 to 0.76 [5,32] in separate cohorts. This difference was not tested within a single cohort and should be interpreted as an indirect, cross-study comparison that is consistent with but does not establish better discrimination by KELIM for this endpoint.

### 3.8. KELIM as a Treatment-Selection Biomarker: Prognostic Versus Predictive Evidence

Beyond prognosis, several studies explored whether KELIM identifies patients likely to benefit from specific treatments. These studies provide three distinct levels of evidence (see Section 2). Predictive evidence, based on a treatment comparison within KELIM strata and a formal KELIM-by-treatment interaction test, was available for three treatments, all from post hoc or observational analyses and none replicated: bevacizumab, in a post hoc analysis of GOG-0218 [2]; weekly dose-dense chemotherapy, in a post hoc analysis of ICON-8 restricted to patients with incomplete surgery [23]; and HIPEC, in a propensity score-matched cohort [28], with a treatment comparison but no interaction test in a second, largely observational study that pointed in the opposite direction [24]. Composite-subgroup evidence, in which outcomes were better with treatment only in patients with unfavorable KELIM combined with suboptimal surgery and no KELIM-by-treatment interaction was tested, was available for atezolizumab [21]. Finally, studies that compared outcomes by KELIM within a single treated population provide prognostic rather than predictive evidence [25,33]. Conventional CA-125 parameters were not evaluated for treatment selection in the included studies, so no comparative statement can be made for this endpoint.

#### 3.8.1. Predictive Evidence: Bevacizumab

In a post hoc analysis of GOG-0218, patients with unfavorable KELIM had longer PFS with bevacizumab (HR: 0.70, 95% CI: 0.59–0.82), whereas those with favorable KELIM did not (HR: 0.96, 95% CI: 0.79–1.17); the KELIM-by-treatment interaction was significant for PFS (*p* = 0.01) but not for OS in the whole population (*p* = 0.08) [2]. A real-world cohort of 114 patients, all of whom received bevacizumab, found longer PFS with favorable KELIM (HR: 0.38, 95% CI: 0.20–0.70) and interpreted this as predicting bevacizumab benefit [33]. Because that cohort had no untreated comparator, its result shows only that KELIM is prognostic among bevacizumab-treated patients; it does not test treatment-effect modification and therefore neither supports nor contradicts the GOG-0218 interaction. The predictive evidence for bevacizumab thus rests on a single post hoc PFS interaction that has not been replicated in another randomized trial, and it does not currently support KELIM-guided selection of bevacizumab.

#### 3.8.2. Predictive Evidence: Dose-Dense Chemotherapy

In a post hoc analysis of ICON-8, weekly dose-dense chemotherapy was associated with longer PFS and OS than three-weekly chemotherapy in the poor-prognosis group defined by unfavorable KELIM and incomplete surgery (HRs of 0.50–0.53 for PFS and 0.57–0.58 for OS) [23]. KELIM-by-treatment interaction tests were reported as significant only among patients with incomplete surgery (PFS: *p* < 0.01 in both the primary- and delayed-surgery cohorts; OS: *p* = 0.03 and *p* = 0.05, respectively) [23]. This is a formal interaction analysis, but it was confined to a surgically defined subgroup, was not prespecified, and was described by its authors as exploratory and hypothesis-generating; it is being tested prospectively in the SALVOVAR trial [23]. It therefore does not establish that KELIM alone should guide the choice of chemotherapy schedule.

#### 3.8.3. Composite-Subgroup Evidence: Atezolizumab

In a post hoc analysis of IMagyn050, a trial in which atezolizumab did not improve PFS overall, PFS was longer with atezolizumab than with a placebo in the poor-prognosis subgroup defined by unfavorable KELIM and suboptimal resection (HR: 0.75, 95% CI: 0.59–0.95) [21]. No KELIM-by-treatment interaction test was reported, and the findings cannot be attributed to KELIM independently of the co-occurring surgical outcome; they should be interpreted as hypothesis-generating.

#### 3.8.4. Predictive Evidence with Discordant Findings: HIPEC

Two NACT-based studies reached opposite conclusions [24,28]. In a pooled analysis of the KOV-HIPEC-01 randomized trial and the prospective KGOG3042 cohort (only 45 of 213 patients from the randomized trial), HIPEC was associated with longer PFS (HR: 0.42) and OS (HR: 0.29) in patients with unfavorable KELIM, with no significant association in those with favorable KELIM, although no formal KELIM-by-HIPEC interaction test was reported [24]. In contrast, a propensity score-matched retrospective cohort reported a significant KELIM-by-HIPEC interaction (*p* = 0.017), in which the HR for HIPEC decreased with increasing KELIM and fell below 1 only above a KELIM value of approximately 1.14, suggesting benefit mainly in patients with favorable KELIM [28]. These directionally opposite findings preclude pooled inference and do not support KELIM-guided selection for HIPEC.

### 3.9. Prognostic Evidence: KELIM and BRCA Status

In a nationwide cohort (*n* = 743), standardized KELIM was higher in BRCA-mutant tumors than in BRCA-wild-type tumors (median: 1.16 vs. 1.06, *p* = 0.001). However, KELIM’s independent prognostic value was retained. The combination of BRCA status and KELIM defined three prognostic groups with progressively divergent PFS (median: 12.0, 16.0–18.8, and 28.8 months) [25], suggesting that KELIM captures chemosensitivity information complementary to, and not fully explained by, BRCA status. Because no treatment comparison was involved, and patients who received PARP inhibitor maintenance were excluded, these data are prognostic and do not inform PARP inhibitor selection.

### 3.10. Risk of Bias

**Figure 7:**
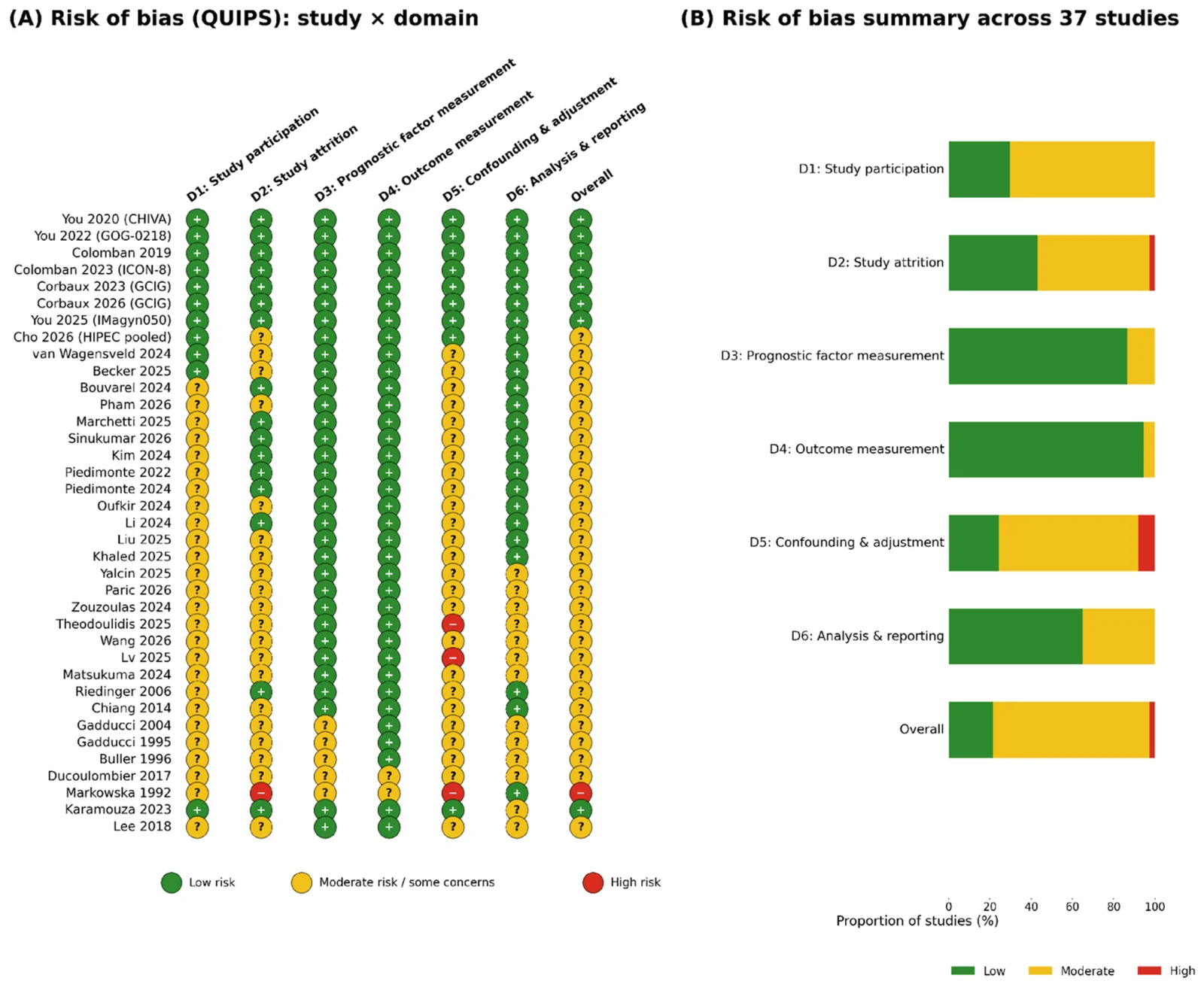
Risk-of-bias assessment (QUIPS) across the 37 included studies. Assessed studies: [2,4,5,6,7,9,11,12,13,14,15,18,19,20,21,22,23,24,25,26,27,28,29,30,31,32,33,34,35,36,37,38,39,40,41,42,43].

Overall, randomized trial sub-analyses and GCIG-IPD meta-analyses were rated as having a low risk of bias, whereas retrospective/real-world cohorts were rated as having a moderate risk, primarily due to their retrospective design and the absence of blinded outcome assessment (Figure 7).

### 3.11. Certainty of Evidence

Funnel plots showed no evident asymmetry, and Egger’s test did not indicate small-study effects for OS (intercept: −0.21, *p* = 0.81) or PFS (intercept: −0.03, *p* = 0.96) (Supplementary Figure S3). Using GRADE, the certainty of evidence was moderate for the association of favorable KELIM with OS and with PFS (downgraded for inconsistency), low for its association with complete cytoreduction (downgraded for risk of bias and inconsistency), and low for the association of CA-125 half-life with OS (downgraded for risk of bias and indirectness, as all three studies were conducted in patients treated with primary surgery between 1995 and 2006). Certainty was very low for the comparative prognostic performance of KELIM versus half-life, because no study evaluated both biomarkers within the same cohort (Supplementary Table S4).

## 4. Discussion

To date, this systematic review and meta-analysis, which was based on a comprehensive formal search, provides the most extensive synthesis of the prognostic value of KELIM in ovarian cancer and updates the indirect comparison of KELIM with conventional CA-125 kinetic parameters across the published literature. Three principal findings emerge.

Compared with the previous meta-analysis by Kim et al. [8], which included literature published before February 2023 and pooled KELIM overall-survival data from five unique studies (seven analyses), the present synthesis adds four elements. First, the evidence base for KELIM and OS has expanded to 14 independent cohorts (approximately 12,900 patients); nine of these cohorts, including the GCIG individual-patient-data meta-analysis and two national registries, were published after that search. The pooled estimate was nearly identical (HR: 0.54 vs. 0.51), indicating that the prognostic association has remained stable as the evidence base has grown and extends to real-world populations. The previous meta-analysis did not rate the certainty of evidence; using GRADE, we rated it as moderate for the association of KELIM with OS and PFS, low for complete cytoreduction, and very low for the comparison with half-life (Supplementary Table S4). Second, the analysis was restricted to first-line treatment, corrected for overlap between the GCIG individual-patient-data estimate and its contributing trials, and explored the sources of heterogeneity, which was attributable mainly to a single registry cohort and to study design; every selected effect estimate was verified against the primary publications under explicit selection rules; the 95% prediction intervals for OS and PFS excluded the null. Third, it provides a pooled estimate for complete cytoreduction after NACT, an outcome that was not synthesized in the previous meta-analysis. Fourth, it distinguishes prognostic from predictive evidence on treatment selection and shows that evidence of differential treatment benefit rests on unreplicated post hoc interaction analyses (bevacizumab and dose-dense chemotherapy) and directionally discordant observational data (HIPEC). In contrast, the comparison with conventional parameters remains unresolved: Kim et al. reported a pooled OS HR of 0.55 (95% CI: 0.32–0.95; I^2^ = 83.5%) for CA-125 half-life from five studies, and our estimate from three studies with adjusted HRs was similar (0.48); neither synthesis therefore supports a clear prognostic advantage of KELIM over half-life, and no head-to-head data have emerged since.

First, favorable KELIM was associated with improved OS (pooled HR: 0.54; 14 independent cohorts after correcting for cohort overlap) and PFS (pooled HR: 0.57; 18 cohorts) in approximately 13,000 patients across randomized trial, GCIG-IPD, and real-world cohorts spanning three continents [2,4,7,25]. The association was weaker in real-world cohorts (HR: 0.63) than in randomized trial data (HR: 0.48), and this difference was statistically significant (*p* = 0.009). This attenuation is expected because real-world populations are unselected, chemotherapy regimens are heterogeneous, and CA-125 sampling is less standardized; it also implies that trial-derived estimates may overstate the prognostic gradient expected in routine practice. Nevertheless, KELIM remained significantly prognostic in real-world cohorts, supporting its generalizability, a prerequisite for clinical adoption.

Second, in this indirect, cross-study comparison, the pooled HR for OS associated with conventional CA-125 half-life was similar in magnitude to that associated with KELIM (0.48 vs. 0.54) [38,40,42]; however, similar HRs or overlapping CIs from different populations do not demonstrate equivalence, and HRs quantify the strength of association rather than discrimination. A conventional CA-125 trajectory model also achieved an AUC of 0.75 for OS in a large GCIG-IPD cohort, although KELIM was not evaluated in that cohort [19]. Thus, the available evidence shows neither that KELIM is superior to conventional CA-125 parameters for prognosis nor that the two are equivalent. KELIM is therefore best regarded as a complementary prognostic measure; half-life or a validated longitudinal CA-125 model may provide similar prognostic stratification, but this has not been tested directly. This interpretation is further limited by era and treatment setting: all three half-life studies enrolled patients treated with primary surgery before NACT and maintenance therapy became routine (see Limitations), so whether half-life performs comparably in contemporary NACT-treated and maintenance-era populations is unknown.

Third, KELIM was associated with surgical outcome, which has not been examined in the same way for conventional CA-125 parameters in the included studies. Favorable KELIM was associated with complete cytoreduction after NACT (pooled OR: 3.34; nine cohorts) [5,7,11,13,14,15,22,29,32]; AUCs for discriminating this endpoint, reported in different cohorts, were 0.71–0.76 for KELIM and ≤0.60 for the conventional CA-125 kinetic models evaluated [18]. A related, non-pooled cohort reported a directionally consistent but non-significant trend: Piedimonte et al. [30], in a cohort designed primarily to correlate KELIM with the histopathologic chemotherapy response score, found a higher rate of no gross residual disease or optimal (<1 cm) cytoreduction with favorable KELIM (97.0% vs. 89.3%; suboptimal cytoreduction 3.0% vs. 10.7%; *p* = 0.08); because this outcome combined complete and optimal resection into a single category rather than isolating complete (0 mm) resection as in the pooled analysis, it was not incorporated into the pooled cytoreduction estimate but is reported narratively as corroborating context. This association concerns the likelihood of a surgical outcome and is prognostic; it does not show that treatment decisions based on KELIM improve survival. For treatment selection, the evidence must be distinguished by type. Better outcomes within a KELIM-defined subgroup do not by themselves establish a differential benefit from a treatment; this requires a comparison between treatments within KELIM strata and a KELIM-by-treatment interaction test. Such analyses were available only for bevacizumab [2] and dose-dense chemotherapy [23], both post hoc analyses of single randomized trials (the latter confined to patients with incomplete surgery), and for HIPEC, for which two largely observational studies reached opposite conclusions [24,28]. The atezolizumab finding derives from a composite subgroup without an interaction test [21], and the findings in a bevacizumab-treated cohort [33] and by BRCA status [25] are prognostic. None of these interactions have been replicated or tested prospectively. Accordingly, the prognostic association of KELIM with survival is established (moderate certainty), whereas its use to select treatment remains exploratory and should not guide clinical decisions outside clinical trials. These are nonetheless the decisions that most directly alter management, and prospective randomized evaluation of KELIM-guided treatment selection is the most important next step.

### 4.1. Limitations

A principal limitation of survival meta-analysis was the observed heterogeneity (OS I^2^ = 72% after correcting for cohort overlap), which was partly explained by study design. Potential sources of residual heterogeneity include varying KELIM cut-offs and contrasts (1.0, 0.925, 0.82, tercile-based) [4,6], diverse treatment settings (PCS vs. NACT, first-line vs. maintenance eras), and mixed histologies. However, exploratory subgroup analyses and meta-regression by KELIM contrast, treatment setting, and treatment era did not identify a significant effect modifier (Supplementary Table S2); the heterogeneity was attributable mainly to a single large registry cohort [7], and these post hoc analyses were underpowered, with one to ten cohorts per subgroup. The cytoreduction analysis (I^2^ = 77%) was additionally affected by small cohorts with sparse cells and by differences in the surgical populations. Accordingly, pooled estimates should be interpreted as broad summaries rather than precise effect sizes. By contrast, conventional CA-125 half-life analysis, albeit based on only three studies [38,40,42], showed no heterogeneity (I^2^ = 0%). However, all three studies were published between 1995 and 2006 and evaluated CA-125 kinetics during chemotherapy given after primary cytoreductive surgery, before NACT became routine and before maintenance bevacizumab or PARP inhibitors were available. In contrast, most KELIM cohorts were treated with NACT (9 of 14 OS cohorts), and KELIM has also been evaluated in bevacizumab-era trials [2] and PARP inhibitor-treated cohorts [22]. This mismatch in era and treatment setting limits the generalizability of the observation that half-life and KELIM had similar pooled HRs in three ways. First, after primary surgery, CA-125 decline during chemotherapy reflects the response of residual disease of variable volume, whereas during NACT it reflects the response of the entire tumor burden; the prognostic value of half-life has not been established in the NACT setting, and the only NACT-based conventional comparator in this review showed poor discrimination for complete cytoreduction (AUC: 0.50–0.57) [18]. Second, maintenance therapies that prolong survival after first-line chemotherapy may modify the association between early CA-125 kinetics and OS, and half-life has not been re-evaluated in such populations. Third, changes in CA-125 assays, sampling schedules, and baseline survival over three decades mean that HRs from historical cohorts may not apply to current practice. The most comparable setting available, KELIM cohorts treated with primary surgery, yielded a similar estimate (pooled HR: 0.50, five cohorts; Supplementary Table S2), but these cohorts were also treated in a different era from the half-life studies. The comparability of half-life and KELIM should therefore be regarded as applying, at most, to historical populations treated with primary surgery and as untested in NACT-treated and maintenance-era populations; a contemporary head-to-head comparison is required.

This study has some limitations. One randomized-trial cohort (Colomban et al. [4] ICON-7) overlapped with the Corbaux et al. [6] GCIG-IPD (MAOV) dataset and was excluded from the primary OS synthesis. Because the trial-level composition of the other GCIG-IPD/IPD meta-analyses included [9,19] was not exhaustively cross-checked against every trial-level sub-analysis in this review, residual undetected overlap cannot be entirely excluded; likewise, overlap between single-center cohorts and multicenter registries could be assessed only from the reported institutions and accrual periods. A re-audit of effect-estimate selection against the primary publications, undertaken during revision, identified errors in the originally submitted analyses: a trial cohort excluded on an incorrect assumption of overlap, a univariable estimate used where a multivariable estimate was available, eligible estimates that had not been pooled, and cytoreduction estimates that did not match their sources. All were corrected under explicit selection rules (Supplementary Table S1), but because these rules were defined after the data had been extracted, they are themselves post hoc, although they were applied uniformly to all studies. Several cytoreduction ORs were unadjusted or calculated from published counts, one rested on very sparse cells [11], and one study reporting R0/R1 rather than complete cytoreduction [12] was not pooled; including it increased the pooled OR to 4.07 (Supplementary Figure S2). In one study, the favorable KELIM group was defined as a score ≤1 [13], the reverse of the convention used elsewhere; its estimates were pooled as reported, and omitting it did not materially change any pooled estimate. The benchmarking of KELIM against conventional CA-125 relied on indirect comparisons among different populations and eras because no study has compared KELIM with CA-125 half-life or nadir within the same cohort; the only within-cohort comparison was with the GCIG CA-125 response criterion [4]. Overlapping confidence intervals and similar AUC ranges in such indirect comparisons do not establish equivalent or superior discrimination, and an OR for complete cytoreduction cannot be compared directly with an AUC reported for another marker. Treatment-selection evidence was synthesized narratively and was graded as exploratory: the only formal KELIM-by-treatment interaction analyses were post hoc analyses of two randomized trials (bevacizumab and dose-dense chemotherapy, the latter confined to patients with incomplete surgery) and observational HIPEC studies with discordant results, and the atezolizumab evidence derives from a composite subgroup only; dedicated, prospectively designed studies are needed before any of these associations can inform treatment selection [24,28]. Several included studies did not contribute to the pooled analyses because they reported only medians or narrative results [34,41,43] or only continuous or composite KELIM estimates (Supplementary Table S1). Several included cohorts were retrospective and rated as having a moderate risk of bias [27,29]. Additionally, KELIM was not established as a surrogate endpoint for survival in the GCIG analysis [6]. Finally, the protocol was registered retrospectively, after the search and screening had been completed; although all post hoc analytic decisions and their corresponding sensitivity analyses are reported, the possibility of selective reporting cannot be fully excluded. In addition, supplementary searching through reference lists and citation chaining is less reproducible than a database search, and the number of records screened through this route was not recorded; however, the two studies identified in this way did not contribute to any pooled estimate.

### 4.2. Future Implications

Future priorities include a prospective head-to-head comparison between KELIM and CA-125 half-life in a single cohort; standardization of KELIM cut-offs; a formal, trial-registry-based overlap assessment across all GCIG-IPD contributing trials; replication of the KELIM-by-bevacizumab and KELIM-by-dose-dense-chemotherapy interactions in independent randomized data, followed by prospective randomized evaluation of KELIM-guided treatment selection; and resolution of conflicting HIPEC data.

## 5. Conclusions

In this systematic review, favorable KELIM was consistently associated with longer overall and progression-free survival across randomized trials and real-world cohorts (pooled HRs: 0.54 and 0.57, respectively), although heterogeneity was substantial. In an indirect, cross-study comparison, conventional CA-125 half-life showed a similar association with overall survival, but these data derive from three older cohorts treated with primary surgery. These findings support the prognostic relevance of early CA-125 kinetics, but they do not establish that KELIM is equivalent or superior to conventional CA-125 assessment, which would require direct within-cohort comparisons. Because it is calculated from routinely measured CA-125 values, KELIM may serve as a complementary approach for monitoring chemosensitivity in patients receiving neoadjuvant chemotherapy. This prognostic association should be distinguished from treatment prediction: evidence that KELIM identifies patients who benefit differentially from specific treatments is limited to unreplicated post hoc interaction analyses and discordant observational data, so its use for treatment selection remains exploratory. Prospective head-to-head studies of prognostic performance and randomized evaluation of KELIM-guided treatment are needed.

## Data Availability

All data analyzed in this review are derived from the published studies included and are presented within this article and its Supplementary Materials. The extraction dataset and analysis scripts are available from the corresponding author on reasonable request.

## Supplementary Materials

The following supporting information can be downloaded at: https://www.mdpi.com/article/10.3390/diagnostics16213474/s1, Search strategy: full PubMed search string; PRISMA 2020 checklist; Table S1: Extraction audit: effect estimate selected for each included study, its source, and reasons for non-pooling; Table S2: Exploration of heterogeneity and sensitivity analyses; Table S3: Records excluded at full-text assessment, with reasons; Table S4: Summary of findings and GRADE certainty of evidence; Figure S1: KELIM and overall survival including the overlapping ICON-7 cohort; Figure S2: KELIM and cytoreduction including the study reporting R0/R1 cytoreduction; Figure S3: Funnel plots for overall and progression-free survival.
