# Supplementary material for: Prognostic Value of the KELIM Score Versus Conventional CA-125 Kinetics in Ovarian Cancer: A Systematic Review and Meta-Analysis
Source: Diagnostics (Basel). 2026 Oct 24;16(21):3474. doi: 10.3390/diagnostics16213474 (PMC13761999; doi:10.3390/diagnostics16213474)

Supplementary Materials

Prognostic Value of the KELIM Score Versus Conventional CA-125 Kinetics in Ovarian Cancer: A Systematic Review and Meta-Analysis

Ying-Hsi Chen and Dah-Ching Ding

Contents

Search strategy: full PubMed search string

PRISMA 2020 checklist

Table S1: Extraction audit: effect estimate selected for each included study, its source, and reasons for non-pooling

Table S2: Exploration of heterogeneity and sensitivity analyses

Table S3: Records excluded at full-text assessment, with reasons

Table S4: Summary of findings and GRADE certainty of evidence

Figure S1: KELIM and overall survival including the overlapping ICON-7 cohort

Figure S2: KELIM and cytoreduction including the study reporting R0/R1 cytoreduction

Figure S3: Funnel plots for overall and progression-free survival

Search string

("Ovarian Neoplasms"[Mesh] OR "epithelial ovarian cancer"[tiab] OR "ovarian carcinoma"[tiab] OR "EOC"[tiab] OR "high grade serous"[tiab] OR "HGSOC"[tiab]) AND ("CA-125 Antigen"[Mesh] OR "CA-125"[tiab] OR "CA125"[tiab] OR "cancer antigen 125"[tiab] OR "KELIM"[tiab] OR "CA-125 elimination rate constant K"[tiab] OR "modeled CA-125 kinetics"[tiab] OR "CA-125 kinetic"[tiab] OR "CA-125 half-life"[tiab] OR "CA-125 nadir"[tiab] OR "longitudinal CA-125"[tiab]) AND ("Antineoplastic Agents"[Mesh] OR "platinum compounds"[tiab] OR "platinum-based chemotherapy"[tiab] OR "carboplatin"[tiab] OR "cisplatin"[tiab] OR "neoadjuvant chemotherapy"[tiab] OR "NACT"[tiab] OR "first-line chemotherapy"[tiab]) AND ("Progression-Free Survival"[Mesh] OR "progression-free survival"[tiab] OR "PFS"[tiab] OR "Survival Analysis"[Mesh] OR "overall survival"[tiab] OR "OS"[tiab] OR "cytoreductive surgery"[tiab] OR "complete cytoreduction"[tiab] OR "optimal debulking"[tiab] OR "prognosis"[Mesh] OR "hazard ratio"[tiab] OR "odds ratio"[tiab] OR "ROC"[tiab] OR "discriminatory"[tiab]) NOT ("Review"[Publication Type] OR "Meta-Analysis"[Publication Type] OR "case reports"[Publication Type])

PRISMA 2020 Checklist

Section and Topic	Item #	Checklist item	Location where item is reported
**TITLE**			
Title	1	Identify the report as a systematic review.	Title
**ABSTRACT**			
Abstract	2	See the PRISMA 2020 for Abstracts checklist.	Abstract
**INTRODUCTION**			
Rationale	3	Describe the rationale for the review in the context of existing knowledge.	Section 1
Objectives	4	Provide an explicit statement of the objective(s) or question(s) the review addresses.	Section 1
**METHODS**			
Eligibility criteria	5	Specify the inclusion and exclusion criteria for the review and how studies were grouped for the syntheses.	Section 2.4
Information sources	6	Specify all databases, registers, websites, organisations, reference lists and other sources searched or consulted to identify studies. Specify the date when each source was last searched or consulted.	Section 2.3
Search strategy	7	Present the full search strategies for all databases, registers and websites, including any filters and limits used.	Section 2.3; Supplementary Materials (search strategy)
Selection process	8	Specify the methods used to decide whether a study met the inclusion criteria of the review, including how many reviewers screened each record and each report retrieved, whether they worked independently, and if applicable, details of automation tools used in the process.	Section 2.5
Data collection process	9	Specify the methods used to collect data from reports, including how many reviewers collected data from each report, whether they worked independently, any processes for obtaining or confirming data from study investigators, and if applicable, details of automation tools used in the process.	Section 2.6
Data items	10a	List and define all outcomes for which data were sought. Specify whether all results that were compatible with each outcome domain in each study were sought (e.g. for all measures, time points, analyses), and if not, the methods used to decide which results to collect.	Section 2.6; Supplementary Table S1
	10b	List and define all other variables for which data were sought (e.g. participant and intervention characteristics, funding sources). Describe any assumptions made about any missing or unclear information.	Section 2.6
Study risk of bias assessment	11	Specify the methods used to assess risk of bias in the included studies, including details of the tool(s) used, how many reviewers assessed each study and whether they worked independently, and if applicable, details of automation tools used in the process.	Section 2.7
Effect measures	12	Specify for each outcome the effect measure(s) (e.g. risk ratio, mean difference) used in the synthesis or presentation of results.	Sections 2.6 and 2.8.1
Synthesis methods	13a	Describe the processes used to decide which studies were eligible for each synthesis (e.g. tabulating the study intervention characteristics and comparing against the planned groups for each synthesis (item #5)).	Sections 2.8.4–2.8.6
	13b	Describe any methods required to prepare the data for presentation or synthesis, such as handling of missing summary statistics, or data conversions.	Sections 2.8.1 and 2.8.3
	13c	Describe any methods used to tabulate or visually display results of individual studies and syntheses.	Section 2.8.12; Table 1; Figures 2–7
	13d	Describe any methods used to synthesize results and provide a rationale for the choice(s). If meta-analysis was performed, describe the	Sections 2.8.1, 2.8.2, 2.8.4

PRISMA

PRISMA 2020 Checklist

Section and Topic	Item #	Checklist item	Location where item is reported
		model(s), method(s) to identify the presence and extent of statistical heterogeneity, and software package(s) used.	and 2.8.11
	13e	Describe any methods used to explore possible causes of heterogeneity among study results (e.g. subgroup analysis, meta-regression).	Sections 2.8.7 and 2.8.8
	13f	Describe any sensitivity analyses conducted to assess robustness of the synthesized results.	Section 2.8.9
Reporting bias assessment	14	Describe any methods used to assess risk of bias due to missing results in a synthesis (arising from reporting biases).	Section 2.8.10
Certainty assessment	15	Describe any methods used to assess certainty (or confidence) in the body of evidence for an outcome.	Section 2.8.10
**RESULTS**			
Study selection	16a	Describe the results of the search and selection process, from the number of records identified in the search to the number of studies included in the review, ideally using a flow diagram.	Section 3.1; Figure 1
	16b	Cite studies that might appear to meet the inclusion criteria, but which were excluded, and explain why they were excluded.	Section 3.1; Supplementary Table S3
Study characteristics	17	Cite each included study and present its characteristics.	Section 3.1; Table 1
Risk of bias in studies	18	Present assessments of risk of bias for each included study.	Section 3.10; Figure 7
Results of individual studies	19	For all outcomes, present, for each study: (a) summary statistics for each group (where appropriate) and (b) an effect estimate and its precision (e.g. confidence/credible interval), ideally using structured tables or plots.	Sections 3.2–3.7; Figures 2–6
Results of syntheses	20a	For each synthesis, briefly summarise the characteristics and risk of bias among contributing studies.	Sections 3.1–3.7; Table 1
	20b	Present results of all statistical syntheses conducted. If meta-analysis was done, present for each the summary estimate and its precision (e.g. confidence/credible interval) and measures of statistical heterogeneity. If comparing groups, describe the direction of the effect.	Sections 3.2, 3.5–3.7; Figures 2 and 4–6
	20c	Present results of all investigations of possible causes of heterogeneity among study results.	Sections 3.3 and 3.4; Figure 3; Supplementary Table S2
	20d	Present results of all sensitivity analyses conducted to assess the robustness of the synthesized results.	Sections 3.2, 3.4, 3.5 and 3.7; Supplementary Figures S1–S2; Supplementary Table S2

PRISMA 2020 Checklist

Section and Topic	Item #	Checklist item	Location where item is reported
Reporting biases	21	Present assessments of risk of bias due to missing results (arising from reporting biases) for each synthesis assessed.	Section 3.11; Supplementary Figure S3
Certainty of evidence	22	Present assessments of certainty (or confidence) in the body of evidence for each outcome assessed.	Section 3.11; Supplementary Table S4
**DISCUSSION**			
Discussion	23a	Provide a general interpretation of the results in the context of other evidence.	Section 4
	23b	Discuss any limitations of the evidence included in the review.	Section 4.1
	23c	Discuss any limitations of the review processes used.	Section 4.1
	23d	Discuss implications of the results for practice, policy, and future research.	Sections 4.2 and 5
**OTHER INFORMATION**			
Registration and protocol	24a	Provide registration information for the review, including register name and registration number, or state that the review was not registered.	Section 2.1
	24b	Indicate where the review protocol can be accessed, or state that a protocol was not prepared.	Section 2.1
	24c	Describe and explain any amendments to information provided at registration or in the protocol.	Section 2.1
Support	25	Describe sources of financial or non-financial support for the review, and the role of the funders or sponsors in the review.	Funding statement
Competing interests	26	Declare any competing interests of review authors.	Conflicts of Interest statement
Availability of data, code and other materials	27	Report which of the following are publicly available and where they can be found: template data collection forms; data extracted from included studies; data used for all analyses; analytic code; any other materials used in the review.	Data Availability Statement; Supplementary Materials

From: Page MJ, McKenzie JE, Bossuyt PM, Boutron I, Hoffmann TC, Mulrow CD, et al. The PRISMA 2020 statement: an updated guideline for reporting systematic reviews. BMJ 2021;372:n71. doi: 10.1136/bmj.n71. This work is licensed under CC BY 4.0. To view a copy of this license, visit https://creativecommons.org/licenses/by/4.0/

Supplementary Table S1. Extraction audit: effect estimate selected for each included study, its source, and reasons for non-pooling

All effect estimates were re-extracted from the primary publications during revision and selected using the rules in Methods (Data Extraction): (i) dichotomous comparison at the standardized KELIM cut-off of 1.0 (or the study-specific dichotomous cut-off), otherwise highest vs lowest category (†); (ii) multivariable (MV) over univariable (UV) estimate; (iii) for complete cytoreduction (no macroscopic residual disease), an OR calculated from published counts when no OR was reported. All estimates are oriented as favorable vs unfavorable KELIM (inverse of the published estimate where the source used unfavorable KELIM as the index group). "Change" describes differences from the originally submitted analysis.

Study [ref]	Cohort(s)	Overall survival: HR (95% CI), source	Progression-free survival: HR (95% CI), source	Complete cytoreduction: OR (95% CI), source	Change on re-audit / reason not pooled
You 2020 [5]	CHIVA phase II RCT, NACT (n=134)	0.28 (0.16–0.50), MV, †highest vs lowest tercile; Table 3	0.36 (0.21–0.62), MV, †tercile; Table 3	13.07 (5.25–37.78), UV, standardized KELIM ≥1.0; Results	Cytoreduction CI corrected (previously 4.87–35.09)
You 2022 [2]	GOG-0218 RCT (n=1,662)	0.55 (0.48–0.62), MV; Results	0.51 (0.46–0.57), MV; Results	Not reported by KELIM group	PFS added (extractable but previously not pooled)
Colomban 2019 [4]	AGO-OVAR-9 (n=1,285); AGO-OVAR-7 (n=189); ICON-7 (n=1,386)	AGO-OVAR-9 0.45 (0.37–0.55); AGO-OVAR-7 0.58 (0.35–0.97); ICON-7 0.44 (0.35–0.53); all MV, †tercile; Table 2	Multivariable PFS estimates reported only in the online supplement; not extracted	Not reported	AGO-OVAR-9 reinstated (previously excluded on an incorrect assumption of overlap with Corbaux 2023); AGO-OVAR-7 added; ICON-7 excluded (included in Corbaux 2023), sensitivity analysis only
Colomban 2023 [23]	ICON-8 RCT (n=1,334)	Not pooled: composite KELIM-and-surgery groups only	Not pooled: as for OS	Not reported	—
Corbaux 2023 [6]	GCIG-MAOV IPD, 8 RCTs; validation set	0.46 (0.41–0.52), MV, validation set; Results	0.49 (0.45–0.54), MV, validation set; Results	Not reported	—
Corbaux 2026 [9]	GCIG-IPD, stage I–II (n=1,143)	0.50 (0.36–0.68), MV; Results	RFS 0.61 (0.48–0.77), MV; Results	Not applicable	Early-stage population: reported separately, not pooled with advanced-stage cohorts
You 2025 [21]	IMagyn050 RCT (n=1,199)	Not reported	0.61 (0.54–0.69), MV; Results	Not reported (composite groups only)	PFS added
Cho 2026 [24]	KOV-HIPEC-01 + KGOG3042, NACT (n=213)	0.53 (0.29–0.98), MV; Table 2	0.57 (0.40–0.79), MV; Table 2	Not reported by KELIM group	OS and PFS added; possible partial overlap with Kim 2024 ICS cohort (sensitivity analysis)
van Wagensveld 2024 [7]	Netherlands registry, NACT (n=1,582)	0.79 (0.69–0.91), MV; Abstract/Results	0.76 (0.66–0.87), MV; Abstract/Results	2.59 (2.04–3.29), UV, favorable vs unfavorable; Results	—
Becker 2025 [25]	ESME registry, NACT	0.54 (0.41–0.70), MV; Table 2	0.56 (0.45–0.68), MV; Table 2	Not reported	CIs corrected (OS upper limit 0.71→0.70; PFS 0.46–0.69→0.45–0.68)
Bouvarel 2024 [15]	Single center, NACT (n=232 with KELIM)	0.67 (0.48–0.93), UV; Table 2	0.68 (0.52–0.91), UV; Table 2	2.32 (1.37–3.96), UV, favorable vs unfavorable; online Supplementary Table 1	OS and PFS added; cytoreduction OR corrected (previous 2.78 [1.30–5.93] did not match the source; 3.87 is the continuous-KELIM OR); possible partial overlap with Becker 2025 (sensitivity analysis)
Pham 2026 [26]	Single center, mostly PCS (n=418)	0.56 (0.37–0.86), MV, inverse of 1.78 (1.16–2.72); Results	Not reported	Not reported	—
Marchetti 2025 [27]	PARPi-treated cohort	Not pooled: KELIM analyzed as continuous variable only	Not pooled: as for OS	Not reported	—

Study [ref]	Cohort(s)	Overall survival: HR (95% CI), source	Progression-free survival: HR (95% CI), source	Complete cytoreduction: OR (95% CI), source	Change on re-audit / reason not pooled
Sinukumar 2026 [28]	HIPEC, propensity-matched	Not pooled: continuous KELIM and KELIM-by-HIPEC interaction only	Not pooled	Not reported	—
Kim 2024 [22]	PCS cohort; ICS cohort (n=252)	Not reported	PCS 0.33 (0.13–0.85), MV, inverse of 3.03 (1.18–7.76); ICS 0.86 (0.43–1.69), MV, inverse of 1.16 (0.59–2.31); Table 2	2.40 (1.05–5.51), counts 38/64 vs 14/37 no residual disease, ICS cohort; Table 1	ICS cohort PFS added (separate patients from PCS cohort)
Piedimonte 2022 [29]	Single center, NACT (n=217)	0.50 (0.25–0.99), MV, inverse of 1.99 (1.01–3.95); Results	0.64 (0.44–0.93), MV, inverse of 1.57 (1.08–2.28); Results	1.30 (0.74–2.31), counts no gross residual 43/76 vs 64/128; Table 1	Cytoreduction previously omitted without explanation; reinstated
Piedimonte 2024 [30]	Single center, NACT (n=172)	Not pooled: composite KELIM-by-CRS groups	Not pooled: as for OS	Not pooled: only optimal (no gross or <1 cm residual) vs suboptimal reported	Excluded by cytoreduction outcome definition
Oufkir 2024 [31]	Single center, NACT (n=149)	Not pooled: no OS HR by KELIM group	Not reported	Not reported by KELIM group	—
Li 2024 [32]	Two centers, NACT (n=133)	0.484 (0.251–0.930), MV, cut-off 0.925; Table 4	0.544 (0.349–0.849), MV; Table 4	5.48 (2.41–12.47), MV; Results	—
Liu 2025 [33]	Bevacizumab-treated cohort (n=114)	Not reported	0.376 (0.202–0.700), MV; Results	Not reported	PFS added
Khaled 2025 [13]	CRS for peritoneal disease, 86% NACT (n=111)	0.561 (0.323–0.976), MV; Table 3	0.683 (0.436–1.071), MV; Table 3	1.36 (0.53–3.48), counts CC-0 32/40 vs 53/71; Results	PFS and cytoreduction added; source defines favorable KELIM as ≤1 (pooled as reported)
Yalcin 2025 [14]	Single center, PDS and IDS (n=102)	Not pooled: OS HR not reported (non-significant in Cox model)	0.44 (0.22–0.88), MV, whole cohort; Results	3.50 (0.37–33.31), counts 5/6 vs 20/34, IDS subgroup; Results	Cytoreduction added
Parić 2026 [34]	Single center (n=92)	Not pooled: medians and P values only	Not pooled: medians only	Not pooled: P value only, no counts in the abstract (full text not available at re-audit)	—
Zouzoulas 2024 [11]	Single center, NACT (n=83)	0.87 (0.25–3.01), MV; Table 5	0.65 (0.26–1.67), MV; Table 4	150 (17.7–1,269), counts 50/51 vs 8/32; Table 2	OS corrected (univariable 0.43 had been used); PFS added; cytoreduction pooled under the outcome-definition rule (previously excluded post hoc for OR >30)
Theodoulidis 2025 [35]	Same institution as [11], NACT (n=78)	0.45 (0.21–0.98), MV; Table 2	0.48 (0.27–0.86), MV; Table 2	Not reported by KELIM group	Overlaps with Zouzoulas 2024 (same department, 2013–2023 vs 2012–2022); smaller cohort, sensitivity analysis only (previously pooled together with [11])
Wang 2026 [12]	Single center, NACT (n=70)	Not pooled: internally inconsistent estimate (OR 0.401, 95% CI 0.317–1.665, P=0.014)	0.519 (0.277–0.973), MV; Results	Not pooled: satisfactory (R0/R1) outcome; inverse of 0.033 (0.008–0.132) used in sensitivity analysis; Table 2	Previously excluded post hoc for OR >30; now excluded by outcome definition
Matsukuma 2024 [36]	Single center (n=55)	Not pooled: association with chemotherapy response score only	Not reported	Not reported	—
Lv 2025 [37]	Single center (n=52)	Not pooled: no OS HR reported	0.25 (0.077–0.818), MV; Abstract	Not reported	—
Ducoulombier 2017 [20]	Single center, NACT (n=54)	Not reported	Not reported	Not pooled: modeled continuous parameter; outcome optimal cytoreduction	—
Riedinger 2006 [38]	CA-125 half-life (n=631)	0.49 (0.38–0.63), MV	—	—	Point estimate and CI not given in

Study [ref]	Cohort(s)	Overall survival: HR (95% CI), source	Progression-free survival: HR (95% CI), source	Complete cytoreduction: OR (95% CI), source	Change on re-audit / reason not pooled
					the abstract; full text not re-checked at revision
Gadducci 1995 [40]	CA-125 half-life (n=225)	0.47 (0.27–0.82), MV	—	—	Point estimate and CI not given in the abstract; full text not re-checked at revision
Gadducci 2004 [42]	CA-125 half-life (n=71)	0.32 (0.13–0.82), MV	—	—	Point estimate matches abstract (inverse of 3.113); CI not given in the abstract

CI, confidence interval; CRS, chemotherapy response score or cytoreductive surgery; HIPEC, hyperthermic intraperitoneal chemotherapy; ICS, interval cytoreductive surgery; IDS, interval debulking surgery; IPD, individual patient data; MV, multivariable; NACT, neoadjuvant chemotherapy; OR, odds ratio; PARPi, poly(ADP-ribose) polymerase inhibitor; PCS/PDS, primary cytoreductive/debulking surgery; RCT, randomized controlled trial; RFS, recurrence-free survival; UV, univariable. Conventional CA-125 studies not listed (Buller, Chiang, Karamouza, Lee, Markowska) were synthesized narratively.

Supplementary Table S2. Exploration of heterogeneity and sensitivity analyses: study-level classification, subgroup and meta-regression analyses, and sensitivity analyses

Subgroup, meta-regression, and sensitivity analyses are post hoc (the subgroup analysis by design was prespecified). Effect estimates are hazard ratios (HRs) or odds ratios (ORs), favorable vs unfavorable KELIM, pooled with a DerSimonian–Laird random-effects model. Subgroup differences were tested with the between-subgroup Q statistic; univariable random-effects meta-regression used a method-of-moments estimator of τ^2.

A. Classification of the 14 cohorts in the primary OS analysis (and the 2 overlapping cohorts used only in sensitivity analyses)

Cohort	Design	KELIM contrast	Treatment setting	Accrual period	Treatment era
**Corbaux 2023 (GCIG-MAOV, 8 RCTs)**	RCT/IPD	Dichotomous ≥1.0	PCS (first-line RCTs)	Before 2019; stated pre-PARPi era	Pre-PARPi
**You 2022 (GOG-0218)**	RCT	Dichotomous ≥1.0	PCS	2005–2009	Pre-PARPi
**Colomban 2019 (AGO-OVAR-9)**	RCT	Tercile	PCS	2002–2004	Pre-PARPi
**Colomban 2019 (AGO-OVAR-7)**	RCT	Tercile	PCS	1999–2004	Pre-PARPi
**You 2020 (CHIVA)**	RCT	Tercile (standardized by 0.07/day)	NACT	2013–2015	Pre-PARPi
**Piedimonte 2022**	Retrospective	Dichotomous ≥1.0	NACT	2010–2019	Extending into PARPi era
**Li 2024**	Retrospective	Dichotomous ≥0.925	NACT	2015–2022	Extending into PARPi era
**van Wagensveld 2024 (Netherlands registry)**	Registry	Dichotomous ≥1.0	NACT	2008–2011	Pre-PARPi
**Bouvarel 2024**	Retrospective	Dichotomous ≥1.0	NACT	2000–2017	Pre-PARPi
**Becker 2025**	Registry	Dichotomous ≥1.0	NACT	2011–2017; 6 PARPi-treated patients excluded	Pre-PARPi
**Khaled 2025**	Retrospective	Dichotomous (≤1 labeled favorable)	NACT (86%)	2010–2021	Extending into PARPi era
**Zouzoulas 2024**	Retrospective	Dichotomous ≥1.0	NACT	2012–2022	Extending into PARPi era

**Cho 2026**	Pooled RCT + cohort	Dichotomous ≥1.0	NACT	Not reported‡	Extending into PARPi era
**Pham 2026**	Retrospective	Dichotomous ≥1.0	PCS (90%)	2015–2019; PARPi not available	Pre-PARPi
**Colomban 2019 (ICON-7)†**	RCT	Tercile	PCS	2006–2007	Pre-PARPi
**Theodoulidis 2025†**	Retrospective	Dichotomous ≥1.0	NACT	2013–2023	Extending into PARPi era

B. Subgroup analyses, meta-regression, sensitivity analyses, and leave-one-out analysis

**Analysis**	**Cohorts**	**Pooled HR or OR (95% CI)**	**I²**	**Subgroup difference**	**Meta-regression: ratio of HRs (95% CI)**
**OS: all cohorts (primary analysis)**	14	0.54 (0.47–0.63)	72%	—	—
**OS design: RCT/GCIG-IPD (prespecified)**	5	0.48 (0.42–0.55)	56%	P=0.009	1.33 (1.08–1.64), P=0.008; R^2 69%
**OS design: retrospective/real-world**	9	0.63 (0.54–0.74)	28%		
**OS contrast: dichotomous ≥1.0**	10	0.58 (0.49–0.69)	75%	P=0.260	Other cut-off 0.83 (0.38–1.83), P=0.652; tercile 0.75 (0.52–1.08), P=0.117; omnibus P=0.280
**OS contrast: dichotomous, other cut-off**	1	0.48 (0.25–0.93)	—		
**OS contrast: highest vs lowest tercile**	3	0.43 (0.32–0.59)	44%		
**OS setting: PCS**	5	0.50 (0.45–0.55)	29%	P=0.265	1.19 (0.93–1.51), P=0.158; R^2 50%
**OS setting: NACT**	9	0.57 (0.46–0.71)	59%		
**OS era: pre-PARPi**	9	0.54 (0.45–0.64)	83%	P=0.982	1.00 (0.67–1.49), P=0.999; R^2 0%
**OS era: extending into PARPi era**	5	0.54 (0.40–0.73)	0%		

**OS sensitivity: including ICON-7**	15	0.53 (0.46–0.61)	72%	—	—
**OS sensitivity: Theodoulidis 2025 replacing Zouzoulas 2024**	14	0.54 (0.46–0.62)	72%	—	—
**OS sensitivity: both Greek cohorts included**	15	0.54 (0.47–0.62)	70%	—	—
**OS sensitivity: excluding Bouvarel 2024 (possible overlap)**	13	0.53 (0.45–0.62)	73%	—	—
**OS sensitivity: excluding tercile-based estimates**	11	0.57 (0.49–0.68)	72%	—	—
**OS leave-one-out: omitting Netherlands registry**	13	0.51 (0.46–0.55)	14%	—	—
**OS leave-one-out: omitting any other single cohort**	13	0.53–0.56	65–74%	—	—
**PFS: all cohorts (primary analysis)**	18	0.57 (0.51–0.63)	62%	—	—
**PFS design: RCT/GCIG-IPD vs retrospective/real-world**	4 / 14	0.52 (0.46–0.59) / 0.61 (0.54–0.69)	70% / 29%	P=0.081	—
**PFS sensitivity: Theodoulidis 2025 replacing Zouzoulas 2024**	18	0.56 (0.51–0.63)	62%	—	—
**PFS sensitivity: excluding possible partial overlaps (Bouvarel 2024; Kim 2024 ICS cohort)**	16	0.55 (0.49–0.62)	63%	—	—
**PFS sensitivity: excluding tercile-based estimate (CHIVA)**	17	0.58 (0.52–0.64)	62%	—	—
**PFS leave-one-out: omitting Netherlands registry**	17	0.54 (0.50–0.59)	28%	—	—
**PFS leave-one-out: omitting any other single cohort**	17	0.56–0.58	52–64%	—	—

**Complete cytoreduction: all cohorts (primary, OR)**	9	3.34 (2.00–5.55)	77%	—	—
**Cytoreduction: excluding Zouzoulas 2024**	8	2.74 (1.80–4.16)	67%	—	—
**Cytoreduction: Haldane–Anscombe correction for Zouzoulas 2024**	9	3.43 (2.05–5.74)	78%	—	—
**Cytoreduction: adding Wang 2026 (R0/R1 outcome)**	10	4.07 (2.36–7.02)	81%	—	—
**Cytoreduction: excluding count-derived ORs from Khaled 2025 and Yalcin 2025**	7	3.80 (2.14–6.75)	82%	—	—
**Cytoreduction leave-one-out**	8	2.74–3.92	67–80%	—	—

KELIM contrast: dichotomous at the standardized cut-off of 1.0; dichotomous at another cut-off; or highest vs lowest tercile (only categorical estimates reported). Treatment era: "pre-PARPi" = accrual completed before 2019 (before approval of first-line PARP inhibitor maintenance) or PARP inhibitors stated to be unavailable; otherwise "extending into PARPi era". Meta-regression ratio of HRs >1 indicates a weaker association in the second-listed category. ‡Accrual period not reported; includes KOV-HIPEC-01 (2010–2016) and the subsequent KGOG3042 cohort; classified as extending into the PARPi era. †Used only in sensitivity analyses (ICON-7: patients included in Corbaux 2023; Theodoulidis 2025: same institution and overlapping accrual period as Zouzoulas 2024, the larger cohort). Possible partial overlap: Bouvarel 2024 (one center contributing to the registry analyzed by Becker 2025) and the interval-surgery cohort of Kim 2024 (may share patients with Cho 2026). CI, confidence interval; IPD, individual patient data; NACT, neoadjuvant chemotherapy; PARPi, poly(ADP-ribose) polymerase inhibitor; PCS, primary cytoreductive surgery; RCT, randomized controlled trial.

Supplementary Table S3. Records excluded at full-text assessment (n = 65), with reasons

Of the 100 reports assessed in full text, 65 were excluded. Exclusion reasons followed PRISMA 2020 principles and are summarized below; the 35 database-derived studies plus 2 hand-searched comparators formed the 37 included studies.

Summary of exclusion reasons: Conference abstract/poster — no full peer-reviewed text (n=49); Does not report a KELIM or CA-125 kinetic biomarker (n=9); Out-of-scope population (recurrent / second-line only) (n=5); Review / secondary study (n=1); Overlapping or duplicate study population (n=1).

No.	Title	Year	PMID
**Conference abstract/poster — no full peer-reviewed text (n = 49)**			
**1**	The predictive and prognostic value of serum CA 125 kinetics and CA 125 nadir during paclitaxel/platinum based chemotherapy in patients with advanced ovarian carcinoma	2009	—
**2**	Dynamic analysis of CA-125 decline during neoadjuvant chemotherapy in patients with epithelial ovarian cancer as a predictor for sensitivity to platinum	2014	—
**3**	The predictive value of the CA-125 modeled kinetic parameter KELIM is validated in 3 independent datasets (AGO-OVAR 7 and 9; ICON 7 AGO/GINECO/GCIG trials)	2016	—
**4**	Two prognostic populations of ovarian cancer patients defined by CA125 modeled kinetic parameter KELIM (AGO-OVAR 7 & 9; ICON 7 AGO/GINECO/ MRC CTU/GCIG trials)	2017	—
**5**	Modeled CA-125 kinetics during neoadjuvant chemotherapy for predicting the likelihood of optimal interval debulking surgery in ovarian cancer patients: data from CHIVA trial (a GINECO study)	2019	—
**6**	Prognostic value and association with veliparib benefit of modeled Ca-125 elimination kinetics (KELIM) in patients with newly diagnosed ovarian cancer: Analysis from the VELIA/GOG-3005 study	2020	—
**7**	The impact of chemosensitivity assessed by modeled CA-125 KELIM on the likelihood of long progression-free survivorship	2020	—

	(PS) after 1st line treatment in ovarian cancer: An analysis of 4,450 patients		
**8**	The prognostic value of chemosensitivity, estimated by the modeled CA-125 KELIM, in ovarian cancer patients treated with neo-adjuvant chemotherapy in the Netherlands	2020	—
**9**	Differential benefit from fractionated dose-dense first-line chemotherapy for epithelial ovarian cancer (EOC) according to KELIM-evaluated tumor primary chemosensitivity: exploratory analyses of ICON-8 trial	2021	—
**10**	Strong association between pathological response to neoadjuvant chemotherapy, tils and modeled Ca125 kelim in ovarian carcinomas: chiva Trial, GINECO	2021	—
**11**	Survival prognostic and surrogate values of the early modeled CA-125 KELIM in newly diagnosed advanced ovarian cancer: Data from the GCIG meta-analysis group	2021	—
**12**	CHEMOSENSITIVITY IN VULNERABLE OLDER PATIENTS IS UNFAVORABLE AND HIGHLY DEPENDENT ON THE TREATMENT REGIMEN: CA125 ELIMINATION RATE CONSTANT K (KELIM) ANALYSIS OF THE GINECO-ENGOT EWOC-1 TRIAL	2022	—
**13**	Long Term Survival of GOG 252 'Randomized Trial of Intravenous Versus Intraperitoneal Chemotherapy plus Bevacizumab in Advanced Ovarian Carcinoma: an NRG Oncology/GOG Study' (021)	2022	—
**14**	The modeled CA-125 elimination rate constant k (KELIM) score as a predictor of treatment response in patients with advanced high grade serous ovarian cancer (190)	2022	—
**15**	A PREDICTIVE VALUE OF CA-125 ELIMINATION OF RATE CONSTANT K(KELIM) ON PROGNOSIS AND DURATION OF BEVACIZUMAB MAINTENANCE THERAPY IN FIRST PLATINUMSENSITIVE RECURRENCE OF OVARIAN CANCER	2023	—
**16**	EXAMINING REAL-	2023	—

	WORLD TREATMENT BENEFITS OF FIRST-LINE MAINTENANCE NIRAPARIB IN NON-BRCA MUTANT HIGH GRADE SEROUS OVARIAN CANCERS		
**17**	KELIM during NEOADJUVANT CHEMOTHERAPY AS PREDICTOR of INTERVAL DEBULKING SURGERY and TREATMENT RESPONSE in ADVANCED OVARIAN CANCER	2023	—
**18**	Prognostic and predictive significance of CA-125 elimination rate constant K (KELIM) in ovarian cancer patients treated with neoadjuvant chemotherapy in a real-world setting	2023	—
**19**	Prognostic role of the modeled CA-125 KELIM in early FIGO stage I and II ovarian cancers (OC): A GCIG individual-patient data meta-analysis	2023	—
**20**	Real-world data from a multi-center study: Insights to the efficacy and safety in patients with ovarian cancer (OC) received niraparib as first-line (1st-L) maintenance therapy (MT)	2023	—
**21**	Strong relationships between the CA-125 KELIM score and the tumor biological effects after neo-adjuvant chemotherapy in advanced ovarian cancer patients: CHIVA trial (GINECO)	2023	—
**22**	Unveiling the KELIM-CRS Connection: Asessing the Correlation in Women with Advanced Ovarian Cancer Undergoing Neoadjuvant Chemotherapy and Interval Debulking Surgery	2023	—
**23**	Utilizing the KELIM score and the chemo-response score to predict chemosensitivity in ovarian cancer (1212)	2023	—
**24**	46MO What are the predictors of the success of interval debulking surgery (CC0-IDS) in patients with advanced ovarian cancers? Consistent data from two large independent datasets	2024	—
**25**	BENEFIT FROM ATEZOLIZUMAB IN PATIENTS WITH A POOR PROGNOSTIC OVARIAN CANCER IN FIRST-LINE SETTING: IMAGYN-050 TRIAL	2024	—
**26**	Baseline value and longitudinal kinetics of	2024	—

	circulating nucleosomes during neoadjuvant chemotherapy in newly diagnosed ovarian cancer: results from the randomized phase II trial CHIVA (GINECO)		
**27**	CA-125 KELIM score predicts progression-free and overall survival in patients with epithelial ovarian cancer undergoing HIPEC at time of interval cytoreductive surgery	2024	—
**28**	Consistent data about the predictive factors of the success of interval debulking surgery (CC0-IDS) in patients with advanced ovarian cancers in two large independent datasets	2024	—
**29**	Correlation of kelim and chemo response grade after neoadjuvant therapy in stage iiic ovarian cancer and its impact as a predictor of survival following interval cytoreduction-bat signal or false alarm?	2024	—
**30**	OUTCOMES OF OVARIAN CANCER SURGERY: PRIMARY VS INTERVAL DEBULKING SURGERY AT HOSPITAL CLINICO SAN CARLOS, MADRID. A RETROSPECTIVE ANALYSIS OF A HIGH-VOLUME GYNAECOLOGICAL CANCER CENTRE IN MADRID (SPAIN)	2024	—
**31**	RELATIONSHIP BETWEEN KELIM SCORE AND PERIOPERATIVE OUTCOMES DURING INTERVAL DEBULKING SURGERY AFTER NEOADJUVANT CHEMOTERAPHY IN ADVANCED OVARIAN CANCER	2024	—
**32**	Role of tumor primary chemosensitivity assessed by modeled CA-125 KELIM to predict complete interval debulking surgery (IDS) in ovarian carcinoma patients treated with neo-adjuvant chemotherapy and immunotherapy: a GINEGEPS/GINECO study of the NeoPembrOv and INeOV trials	2024	—
**33**	THE VALUE OF KELIM IN PREDICTING COMPLETE RESECTION AFTER NEOADJUVANT CHEMOTHERAPY IN ASIAN POPULATION WITH ADVANCED	2024	—

	EPITHELIAL OVARIAN CANCER		
**34**	VALIDITY OF CA-125 ELIMINATION RATE CONSTANT K FOR INDICATING PROGNOSIS AND THERAPEUTIC SELECTION AMONG JAPANESE PATIENTS WITH HIGH-GRADE SEROUS OVARIAN CARCINOMA: A SINGLE-CENTER RETROSPECTIVE STUDY	2024	—
**35**	Assessing the relationship between KELIM score and response to niraparib first-line maintenance therapy in non-BRCA mutant advanced ovarian cancer	2025	—
**36**	Difference Of Chemosensitivity Between Western And Japanese Women In First-Line Setting Of Advanced Serous Ovarian Cancer: Data From The MAOV Individual Patient Data Meta-Analysis	2025	—
**37**	KELIM As A Marker Of Chemosensitivity In Patients With Advanced Ovarian Cancer After Neoadjuvant Chemotherapy	2025	—
**38**	KELIM Score As A Predictive And Prognostic Marker Of Platinum Sensitivity In Locally Advanced Epithelial Ovarian Cancer: Egyptian Cancer Center Experience	2025	—
**39**	PREDICTIVE MARKERS OF RESPONSE TO ADJUVANT TREATMENT IN LOCALLY ADVANCED OVARIAN CANCER	2025	—
**40**	Rationale and study design of the KOV-HIPEC-04 trial: a phase III randomized controlled trial in primary stage III and IV ovarian cancer after interval cytoreductive surgery (FOCUS)	2025	—
**41**	The Prognostic Role Of Kelim In Recurrent Ovarian CAncer: From CA 125 Kinetics To SurvivaL DatA (KOALA Study)	2025	—
**42**	109RO Continuous relationship between KELIM chemosensitivity and survival benefit from chemotherapy intensification (standard or dose-dense; with/without bevacizumab): ICON8 & 8B	2026	—
**43**	227eP Prognostic value of the modeled CA-125 elimination constant K (KELIM) score and its correlation with	2026	—

	relapse-free survival in high-grade serous ovarian carcinoma		
**44**	Association of adjuvant chemotherapy regimen with serial CA-125 kinetics (KELIM framework) and progression-free survival after cytoreductive surgery for ovarian cancer: A retrospective cohort study	2026	—
**45**	Dynamic ctDNA and CA-125 kinetics during neoadjuvant chemotherapy as predictors of interval cytoreduction and progression-free survival in high-grade serous ovarian cancer: A systematic review and meta-analysis	2026	—
**46**	Evaluating the CA-125 KELIMTM score across racial groups as a predictor of chemotherapy response and survival in advanced epithelial ovarian cancer	2026	—
**47**	Prognostic impact of carboplatin-paclitaxel versus nedaplatin-paclitaxel in advanced epithelial ovarian cancer - A real world study from single cancer center	2026	—
**48**	The influence of KELIM score on the treatment decisions for patients with advanced epithelial ovarian cancer	2026	—
**49**	The prognostic value of CA125 KELIM in advance stage ovarian cancer undergoing neoadjuvant chemotherapy: a systematic review and meta-analysis	2026	—
**Does not report a KELIM or CA-125 kinetic biomarker (n = 9)**			
**50**	Evaluation of the Time-Varying Effect of Prognostic Factors on Survival in Ovarian Cancer.	2015	25777089
**51**	Prognostic analysis of peritoneal washing cytology during interval debulking surgery in advanced ovarian cancer.	2024	39182152
**52**	The Influence of Circulating Immune Cell and CA125 Dynamics on Neoadjuvant Therapy Selection for Advanced Ovarian Cancer.	2024	39202571
**53**	Enhancing Prognosis in Advanced Ovarian Cancer: Primary Cytoreductive Surgery and Adjuvant Chemotherapy or Neoadjuvant Chemotherapy and Interval Cytoreduction-A Single-Center Retrospective Observational Study.	2025	40282490
**54**	Pretreatment circulating tumor DNA and tissue	2025	41362586

	genomic profiling concordance in ovarian cancer: a prospective observational study of influencing factors and clinical implications.		
**55**	Exploratory analysis of serial pan-immune-inflammation value and its early dynamics during neoadjuvant chemotherapy as a predictor of complete cytoreduction at interval debulking surgery in advanced ovarian cancer: a retrospective cohort study.	2026	42001161
**56**	Multimodal data-based graph convolutional networks for predicting outcomes in ovarian cancer receiving neoadjuvant chemotherapy.	2026	41792435
**57**	Neoadjuvant Bi-Directional Chemotherapy with PIPAC and Systemic Chemotherapy in Advanced Epithelial Ovarian Cancer: An Emerging Conversion Strategy for Initially Unresectable Peritoneal Disease.	2026	42023184
**58**	Prognostic Nomogram for Ovarian Cancer Patients on First-Line Maintenance Therapy With PARP Inhibitors: A Retrospective Cohort Study.	2026	42169627
**Out-of-scope population (recurrent / second-line only) (n = 5)**			
**59**	The strong prognostic value of KELIM, a model-based parameter from CA 125 kinetics in ovarian cancer: data from CALYPSO trial (a GINECO-GCIG study).	2013	23694718
**60**	KELIM score predicts outcome in patients with platinum-resistant/refractory recurrent ovarian cancer.	2023	37309756
**61**	Bevacizumab, olaparib, and durvalumab in patients with relapsed ovarian cancer: a phase II clinical trial from the GINECO group.	2024	38443333
**62**	Extending the timing for secondary cytoreductive surgery after second-line chemotherapy in relapsed ovarian cancer: the EXTENSION study.	2025	40328635
**63**	Prognostic Value of a Joint K-PD Model With Tumor Size Dynamics and CA-125 Kinetics in Recurrent Ovarian Cancer Patients: BOLD Phase II GINECO Study.	2025	40622844
**Review / secondary study (n = 1)**			
**64**	Prognostic value of CA125 kinetics, half-life, and nadir in the treatment of epithelial ovarian cancer: a systematic	2023	37949486

	review and meta-analysis.		
**Overlapping or duplicate study population (n = 1)**			
**65**	Early Clinical and Molecular Biomarkers in Patients With Advanced Ovarian Cancer Undergoing Neoadjuvant Chemotherapy: CHIVA Phase II GINECO Trial.	2026	41533997

PMID = PubMed identifier; "—" indicates no PMID (conference abstracts/posters not indexed in PubMed). Records identified and de-duplicated with Rayyan; full-text eligibility assessed against the pre-specified inclusion criteria.

Supplementary Table S4. Summary of findings and GRADE certainty of evidence

Certainty of evidence was rated post hoc using the GRADE approach for prognostic factor research (Foroutan et al., J Clin Epidemiol 2020). Because the question concerns the association between a prognostic factor and outcomes in cohorts (including post hoc analyses of randomized trials analyzed as cohorts), evidence started at high certainty and was downgraded for risk of bias, inconsistency, indirectness, imprecision, and publication bias.

**Outcome / comparison**	**Cohorts (patients*)**	**Pooled estimate (95% CI)**	**I^2 / 95% PI**	**Risk of bias**	**Inconsistency**	**Indirectness**	**Imprecision**	**Publication bias**	**Certainty**
**KELIM (favorable vs unfavorable) – overall survival**	14 (12,938)	HR 0.54 (0.47–0.63)	72% / 0.34–0.87	Not serious†	Serious (−1)‡	Not serious	Not serious	Not detected (Egger P = 0.81)	⊕⊕⊕◯ Moderate
**KELIM (favorable vs unfavorable) – progression-free survival**	18 (12,835)	HR 0.57 (0.51–0.63)	62% / 0.41–0.79	Not serious†	Serious (−1)‡	Not serious	Not serious	Not detected (Egger P = 0.96)	⊕⊕⊕◯ Moderate
**KELIM (favorable vs unfavorable) – complete cytoreduction**	9 (2,898)	OR 3.34 (2.00–5.55)	77% / 0.68–16.36	Serious (−1)§	Serious (−1)‖	Not serious	Not serious	Not assessable (<10 cohorts)	⊕⊕◯◯ Low
**CA-125 half-life (short vs long) – overall survival**	3 (927)	HR 0.48 (0.38–0.60)	0% / –	Serious (−1)¶	Not serious	Serious (−1)#	Not serious	Not assessable (<10 cohorts)	⊕⊕◯◯ Low
**KELIM vs CA-125 half-life – comparative prognostic performance (OS)**	No within-cohort comparison	Not estimable (indirect: HR 0.54 vs 0.48)	–	Serious (−1)	Not serious	Very serious (−2)**	Not serious	–	⊕◯◯◯ Very low

*Sum of the study sample sizes in Table 1; analysis-specific numbers may be smaller. †Most retrospective cohorts were at moderate risk of bias, but the association was present in the low-risk randomized-trial/GCIG-IPD subgroup (OS HR 0.48; PFS HR 0.52) and remained significant, though weaker, in real-world cohorts (OS HR 0.63, P for difference = 0.009; PFS HR 0.61, P = 0.081); because restriction to low-risk studies did not weaken the association, not downgraded. ‡Moderate-to-substantial statistical heterogeneity, although all cohort point estimates favored favorable KELIM and the 95% prediction interval excluded 1; heterogeneity was attributable mainly to one registry cohort and to study design. §Eight of nine cohorts were observational analyses; several ORs were unadjusted or calculated from published counts, and one rested on very sparse cells (pooled OR excluding it 2.74; adding a study reporting R0/R1 cytoreduction 4.07). ‖Substantial heterogeneity with a 95% prediction interval crossing 1. ¶All studies were retrospective with moderate risk of bias. #Studies published 1995–2006 in patients treated with chemotherapy after primary surgery, before routine NACT and maintenance therapy; applicability to current practice is limited. **No study evaluated both biomarkers in the same cohort; comparison based on different populations, treatment settings, and eras. CI, confidence interval; GCIG-IPD, Gynecologic Cancer InterGroup individual patient data; HR, hazard ratio; NACT, neoadjuvant chemotherapy; OR, odds ratio; OS, overall survival; PI, prediction interval.

Supplementary Figure S1. KELIM score (favorable vs unfavorable) and overall survival — sensitivity analysis including the overlapping ICON-7 cohort (15 cohorts)

This figure adds the Colomban et al. (2019) ICON-7 cohort (orange) to the 14 independent cohorts of the primary analysis (main text, Figure 2). ICON-7 is one of the eight randomized trials whose individual patient data underlie the Corbaux et al. (2023) GCIG-IPD (MAOV) estimate, so its inclusion double-counts these patients; the AGO-OVAR-9 and AGO-OVAR-7 cohorts reported by Colomban et al. are not part of that dataset and are included in the primary analysis. DerSimonian–Laird random-effects model. Pooled HR 0.53 (95% CI 0.46–0.61), $I^2 = 72\%$, compared with 0.54 (0.47–0.63), $I^2 = 72\%$ in the primary analysis. †Highest versus lowest KELIM tercile (no dichotomous estimate reported).

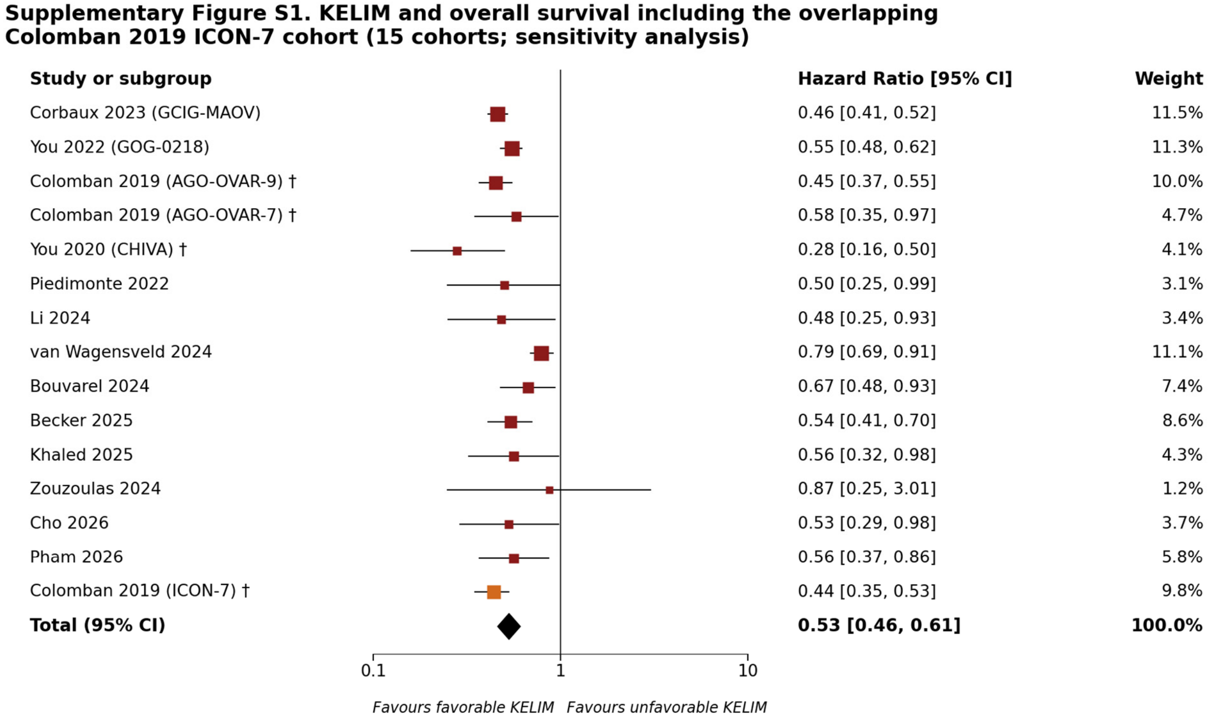

Heterogeneity: $Tau^2 = 0.041$; $I^2 = 72\%$; Q = 50.7, df = 14; 95% prediction interval 0.33–0.84

Test for overall effect: Z = 8.84 (P < 0.0001)

† Highest vs lowest KELIM tercile (no dichotomous ≥1.0 estimate reported).

ICON-7 (orange) is also one of the eight trials in the Corbaux 2023 GCIG-MAOV dataset; its inclusion double-counts these patients.

Supplementary Figure S2. KELIM score (favorable vs unfavorable) and cytoreduction — sensitivity analysis adding the study reporting satisfactory (R0/R1) cytoreduction (10 cohorts)

The primary analysis (main text, Figure 6) pools the nine cohorts reporting complete cytoreduction (no macroscopic residual disease). Wang et al. (2026) reported satisfactory (R0/R1) cytoreduction and is therefore not eligible under the outcome definition; this figure adds it (orange), using the inverse of its published multivariable OR (0.033, 95% CI 0.008–0.132, reported with unfavorable KELIM as the index group; inverted OR 30.30, 95% CI 7.58–125.00). DerSimonian–Laird random-effects model. Pooled OR 4.07 (95% CI 2.36–7.02), I^2 = 81%, compared with 3.34 (2.00–5.55), I^2 = 77% in the primary analysis. Other cytoreduction sensitivity analyses (Supplementary Table S2): excluding Zouzoulas et al. (2024), whose OR of 150 rests on one incomplete resection among 51 patients with favorable KELIM, OR 2.74 (1.80–4.16), I^2 = 67%; Haldane–Anscombe continuity correction for Zouzoulas et al. (OR 97.0, 16.0–588.0), pooled OR 3.43 (2.05–5.74).

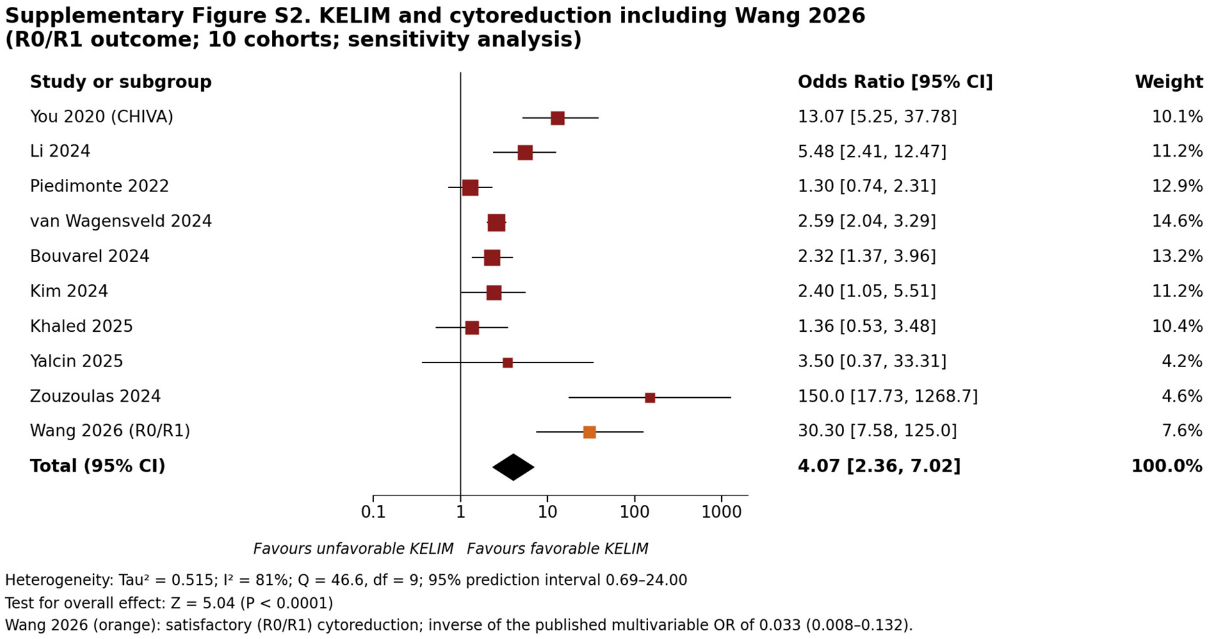

Supplementary Figure S3. Funnel plots for the KELIM syntheses of overall and progression-free survival

Funnel plots of the log hazard ratio against its standard error for (A) overall survival (14 cohorts) and (B) progression-free survival (18 cohorts). The vertical solid line indicates the pooled random-effects estimate, and the dashed lines indicate the pseudo-95% confidence limits. Egger's regression test showed no evidence of small-study effects (overall survival: intercept −0.21, P = 0.81; progression-free survival: intercept −0.03, P = 0.96). Funnel plot asymmetry was not assessed for outcomes with fewer than 10 cohorts.

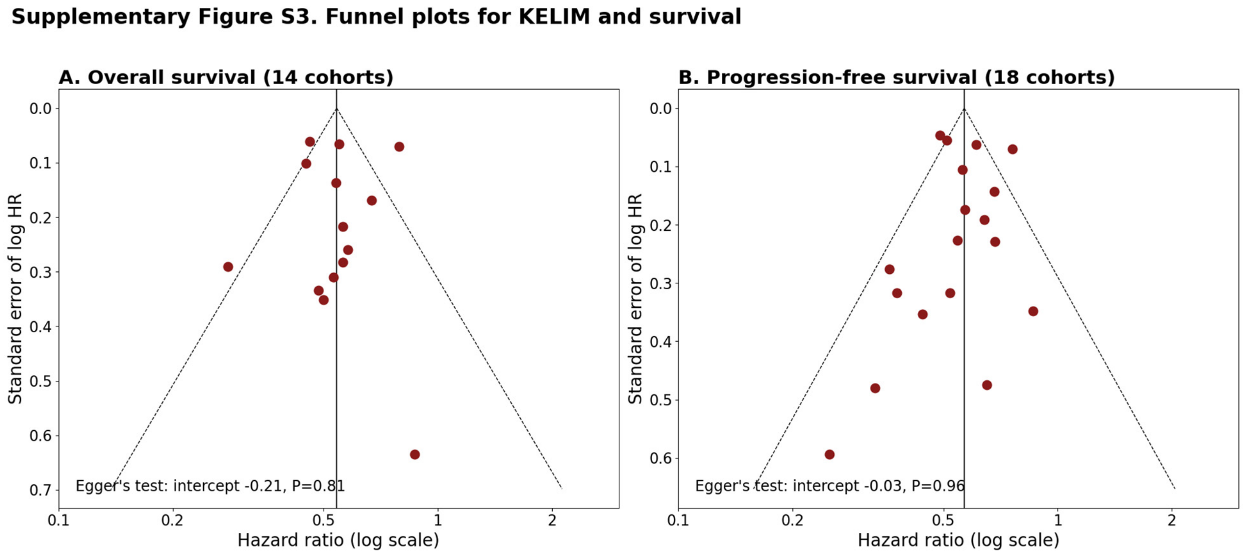

Supplement: Supplementary file 1 [file diagnostics-16-03474-s001.zip › diagnostics-4612758-supplementary.pdf]
